# SH3GLB1-related autophagy mediates mitochondrial metabolism to acquire resistance against temozolomide in glioblastoma

**DOI:** 10.1186/s13046-022-02429-8

**Published:** 2022-07-13

**Authors:** Chia-Hung Chien, Wen-Bin Yang, Jian-Ying Chuang, Jung-Shun Lee, Wei-An Liao, Chih-Yuan Huang, Pin-Yuan Chen, An-Chih Wu, Shun-Tai Yang, Chien-Cheng Lai, Pei-I Chi, Jui-Mei Chu, Siao Muk Cheng, Chan-Chuan Liu, Daw-Yang Hwang, Shang-Hung Chen, Kwang-Yu Chang

**Affiliations:** 1 National Institute of Cancer Research, National Health Research Institutes, Tainan, Taiwan; 2 School of Medicine, I-Shou University, Kaohsiung, Taiwan; 3 TMU Research Center of Neuroscience, Taipei Medical University, Taipei, Taiwan; 4 The Ph.D. Program for Neural Regenerative Medicine, College of Medical Science and Technology, Taipei Medical University, Taipei, Taiwan; 5 Department of Biomedical Science and Environmental Biology, Kaohsiung Medical University, Kaohsiung, Taiwan; 6 Division of Neurosurgery, Department of Surgery, National Cheng Kung University Hospital, College of Medicine, National Cheng Kung University, Tainan, Taiwan; 7 Department of Cell Biology and Anatomy, College of Medicine, National Cheng Kung University, Tainan, Taiwan; 8 Department of Pathology, National Cheng Kung University Hospital, College of Medicine, National Cheng Kung University, Tainan, Taiwan; 9 Department of Neurosurgery, Chang Gung Memorial Hospital at Keelung, Keelung, Taiwan; 10 School of Medicine, Chang Gung University, Taoyuan, Taiwan; 11 Department of Neurosurgery, Chang Gung Memorial Hospital at Linkou, Taoyuan, Taiwan; 12 Graduate Institute of Medical Sciences, College of Medicine, Taipei Medical University, Taipei, Taiwan; 13 Division of Neurosurgery, Shuang-Ho Hospital, Taipei Medical University, Taipei, Taiwan; 14 Department of Oncology, National Cheng Kung University Hospital, College of Medicine, National Cheng Kung University, Tainan, Taiwan

**Keywords:** SH3GLB1, Temozolomide, Resistance, Mitochondrial functions, Autophagy

## Abstract

**Background:**

The mechanism by which glioblastoma evades temozolomide (TMZ)-induced cytotoxicity is largely unknown. We hypothesized that mitochondria plays a role in this process.

**Methods:**

RNA transcriptomes were obtained from tumor samples and online databases. Expression of different proteins was manipulated using RNA interference or gene amplification. Autophagic activity and mitochondrial metabolism was assessed in vitro using the respective cellular and molecular assays. In vivo analysis were also carried out in this study.

**Results:**

High SH3GLB1 gene expression was found to be associated with higher disease grading and worse survival profiles. Single-cell transcriptome analysis of clinical samples suggested that SH3GLB1 and the altered gene levels of oxidative phosphorylation (OXPHOS) were related to subsets expressing a tumor-initiating cell signature. The SH3GLB1 protein was regulated by promoter binding with Sp1, a factor associated with TMZ resistance. Downregulation of SH3GLB1 resulted in retention of TMZ susceptibility, upregulated p62, and reduced LC3B-II. Autophagy inhibition by SH3GLB1 deficiency and chloroquine resulted in attenuated OXPHOS expression. Inhibition of SH3GLB1 in resistant cells resulted in alleviation of TMZ-enhanced mitochondrial metabolic function, such as mitochondrial membrane potential, mitochondrial respiration, and ATP production. SH3GLB1 modulation could determine tumor susceptibility to TMZ. Finally, in animal models, resistant tumor cells with SH3GLB1 knockdown became resensitized to the anti-tumor effect of TMZ, including the suppression of TMZ-induced autophagy and OXPHOS.

**Conclusions:**

SH3GLB1 promotes TMZ resistance via autophagy to alter mitochondrial function. Characterizing SH3GLB1 in glioblastoma may help develop new therapeutic strategies against this disease in the future.

**Supplementary Information:**

The online version contains supplementary material available at 10.1186/s13046-022-02429-8.

## Background

Glioblastoma (GBM) is the most common primary malignant brain tumor, with an annual age-adjusted incidence rate of 3.19 per 100,000 populations in the US. GBM’s prognosis is disappointing, with a 2-year overall survival rate of only 13.6% [1]. Currently, temozolomide (TMZ) is the most frequently administered alkylating agent for patients with GBM [2]. However, the survival benefit from this chemotherapeutic agent is only modest, as TMZ-taking patients only show a 2.5 months longer of survival compared to those without taking TMZ [3]. One of the common causes leading to treatment failure is the development of resistance. In addition to the mechanism associated with O^6^-methylguanine-DNA methyltransferase and other DNA-repairing genes [4], our previous study revealed that upregulation of superoxide dismutase 2 (SOD2) enhanced cellular tolerance to reactive oxygen species (ROS), promoting the induction of TMZ resistance [5]. This led to enrichment of specific subsets carrying tumor-initiating cell (TIC) features against the antitumor drug. It also highlighted the ability for tumors to adapt when they encountered stressful environment.

SOD2 functions in mitochondria to modulate intracellular oxidative stress, and thereby regulates cell metabolism [6]. Recent studies have suggested that altered metabolic reprogramming by mitochondria promotes the induction of chemotherapy resistance in cancer cells [7]. In GBM, the altered metabolic reprogramming and the association with the induction of TMZ resistance has likewise been reported [8]. Rabé and colleagues suggested that a transient increase in the oxygen consumption rate (OCR)/extracellular acidification rate (ECAR) ratio might cause cellular resistance to TMZ [9]. However, the promoting factors remain not clear. Autophagy is a regulator of oxidative phosphorylation (OXPHOS) [10]. Autophagy degrades and clears the aggregated proteins and dysfunctional organelles to maintain cellular homeostasis [11]. Upregulation of autophagy can promote either the survival or death of cancer cells, depending on the stimulation received. For example, in a tumor environment with limited nutrients and oxygen, an increase in autophagy allows cancer cells to survive and resume proliferation and initiation [12]. In GBM, certain subsets, particularly those with the capability of self-renewal and differentiation, activate autophagy to survive TMZ treatment and the harsh microenvironment [13].

Among different autophagy regulators, SH3GLB1 (Bax-Interacting Factor 1 or endophilin B1) is indispensable for the initiation of autophagy [14]. This evolutionarily conserved protein is a member of the endophilin protein family, which contains an N-terminal BAR domain and a C-terminal SH3 domain [15]. It recruits beclin1 and activates PI3KC3, which is the crucial step for dimerization and interaction with ultraviolet irradiation resistance–associated gene in forming the early autophagosome [16]. Interestingly, the functional impact of SH3GLB1 is broad and profound, including participating in the processes of mitochondrial dynamics, apoptosis, and endocytosis in membrane reshaping to maintain intracellular homeostasis [14]. Notably, SH3GLB1 is physiologically important for maintaining the function of brain, as it protects neuronal cells from amyloid-β-induced cytotoxicity to induce the progression of Alzheimer’s disease [17].

Given that altered SOD2 and mitochondrial functions promote resistance in GBM, it remains unknown how the related metabolic reaction contributes to the process. Considering that SH3GLB1 is fundamental in mitochondrial function [18], we hypothesized that SH3GLB1 associates with SOD2 to steer the organelle response against TMZ in GBM. Here, we demonstrated that enhanced SH3GLB1 expression was common in recurrent tumors. The protein modulated autophagy and OXPHO to promote TMZ resistance.

## Materials and methods

### Cell culture

Human GBM cell lines U87MG and A172 (American Type Culture Collection; Manassas, VA, USA), as well as Pt#3 and Pt#5, which both were derived from GBM patients [19], were cultured in 10% fetal bovine serum and antibiotic-supplemented DMEM (Thermo Fisher Scientific). Resistant cells (U87MG-R, A172-R, Pt#3-R, and Pt#5-R) were developed and selected from parental cells by using prolonged TMZ treatment as our previous studies [5, 20]. A primary GBM tumor P1S, which was obtained from a GBM tissue that exhibited therapeutic resistance, was maintained as a patient-derived xenograft in immunodeficient mice before cryopreservation [19].

### PCR array

The mRNA of the cells was extracted and processed to cDNA, following by analysis of mitochondria-related genes with RT^2^ Profiler™ PCR Array Human Mitochondria (#PAHS-087Z, Qiagen, Hilden, Germany).

### Analysis of clinical datasets

Online GBM databases from GlioVis for survival [21] and from the Chinese Glioma Genome Atlas (CGGA) [22] for assessment of clinical parameter was utilized to reveal the impact of genes in interest. The data of RSEM (RNA-Seq by Expectation Maximization) values were downloaded for further processing. The expression fold change and significance level (*p*-value) of mitochondria-related gene expression between primary and resistant GBM RNA-sequencing data were calculated.

### Preparation of clinical samples and immunohistochemistry (IHC)

Nine paired primary and recurrent tumor sections were prepared from archived formalin-fixed, paraffin-embedded blocks with their clinical information blind to investigators. All were diagnosed after 2011 and were histologically confirmed for GBM. A DAKO IHC kit (Agilent Technologies, Santa Clara, CA, USA) was used following the manufacturer’s protocol. The staining results of the clinical samples were assessed by a neuropathologist.

### Treatment reagents and detecting antibodies

TMZ, mithramycin A, and chloroquine were purchased from Sigma–Aldrich (St. Louis, MO, USA). The detection antibodies were as follows: SH3GLB1 (Proteintech, Rosemont, IL, USA), CD133 (Proteintech), SP1 (Merck Millipore, Burlington, MA, USA), LC3B (Santa Cruz, Dallas, TX, USA), p62 (Cell Signaling, Danvers, MA, USA), caspase 3 (Cell Signaling), actin (Merck Millipore), Atg12 (GeneTex, Irvine, CA, USA), and OXPHOS antibody cocktail (ab110411, Abcam, Cambridge, UK), Vinculin (Thermo Fisher Scientific).

### Whole genome RNA sequencing

The 14 paired recurrent and primary frozen samples of high-grade glioma samples were obtained. Total RNA was extracted and quantified following the manufacturer’s protocol (TRIzol; Thermo Fisher Scientific, MA, USA). Strand-Specific RNA Library Kit (Agilent Technologies, Inc., CA, USA) was prepared for *mRNA sequencing* by *Illumina* system and AMPure XP magnetic beads (Beckman Coulter, CA, USA) were used for purification. For genetic analysis based on molecular functioning, we applied Ingenuity Pathway Analysis (IPA, Qiagen, Hilden, Germany) on the sequencing data.

### Single-cell RNA (scRNA) transcriptomics

Inform consents were given to newly diagnosed GBM patients that were planned for surgical resection to obtain tumor tissues for study. Samples were dissociated using a Brain Tumor Dissociation Kit, papain (Miltenyi Biotec, Bergisch Gladbach, Germany) and ACK RBC Lysing Buffer (Thermo Fisher Scientific). An scRNA library was then constructed by generating gel beads in emulsion (GEM) using a Chromium Next GEM Single Cell 3’GEM, Library & Gel Bead Kit (v3.1; 10X Genomics, Pleasanton, CA, USA). The gel beads were then dissolved, primers were released, and the co-partitioned cells were lysed. cDNA was then synthesized, followed by purification with magnetic bead, barcoding, and amplification by PCR for library construction. RNA sequencing (RNA-seq) was performed using Illumina next-generation sequencing platforms.

For genetic sorting of the scRNA transcriptomics, the raw data were aligned to the GRCh38 reference genome, namely and output into aggregated files that integrated individual cells with their annotated information via CellRanger 4.0 (10X Genomics). Analysis was then performed using Loupe Browser 5.0 (10X Genomics), with the criteria set to exclude low complexity cells (< 1000 genes, < 1800 UMI), high complexity cells (> 6500 genes), and dying cells (> 12% UMI to mitochondrial genes) [23].

For analysis, hierarchical clustering was performed using the Euclidean distance method, and average linkage was determined according to the log2 (fold change) values of the OXPHOS genes [24]. Comparative analysis (similarity matrix) between each cluster were generated using Morpheus (https://software.broadinstitute.org/morpheus). The scores in the similarity matrix were calculated using the Pearson correlation coefficient.

### RNA-based gene modulation

Lipofectamine® RNAiMAX reagent (Invitrogen) was used for the transfection of small interfering RNA (siRNA) targeting SH3GLB1 (5′-GGGAAUCAGCAGUACACAUTT-3′ and 3′-AUGUGUACUGCUGAUUCCCTT-5′, GenePharma, *Shanghai, China*), Sp1 (ON-TARGETplus siRNA; Horizon Discovery, Cambridge, UK), and the control. Long-term modulation was achieved by selecting clones for lentiviral-transfected cells using SH3GLB1 short-hairpin RNA (shRNA; RNAi Core of Academia Sinica, Taipei, Taiwan) or the overexpression vector (GenScript Biotech, NJ, USA).

### Western blot analysis

Cell lysates were separated using sodium dodecyl sulfate–polyacrylamide gel electrophoresis and then transferred onto polyvinylidene difluoride membranes (Bio-Rad, Hercules, CA, USA). The membranes were blocked using 5% nonfat milk and incubated overnight with primary antibodies, and labeled with secondary antibodies. A chemiluminescence substrate was used to elicit signals for detection of the intensity.

### Cell density assay

For the cell density assay, 5000–20,000 cells were plated in a 24-well plates under treatment until they stabilized. They were stained with 50% ethanol containing 0.5% methylene blue for 90 min and dissolved in 1% N-lauroylsarcosine for measurement with optical density at 570 nm.

### Sorting of CD133^+^ cells

Patient-derived xenograft tumor cells were used as primary cell cultures. They were cultured in serum-free growth factor-supplemented DMEM/F12 (Thermo Fisher Scientific). The cells were collected and disaggregated with repeat pipetting. They were than labeled with fluorochrome-conjugated anti-CD133 antibodies (Miltenyi Biotec) and were sorted by FACSAria™ III (BD Biosciences). The isolated CD133^+^ cells were then cultured in growth factor-supplemented DMEM/F12. Simultaneously isolated CD133^−^ cells were cultured in serum-supplemented DMEM to maintain the differentiation phenotype.

### Fluorescent labeling of autophagy

Cells were transfected with a control vector or LC3B-EGFP plasmid (courtesy of Chun Hei Antonio Cheung, National Cheng Kung University, Tainan, Taiwan) using Lipofectamine LTX reagent (Invitrogen) to label autophagosomes. Autolysosomes were stained with acridine orange (Sigma–Aldrich), which is a cell-permeable green fluorophore that shifts to red fluorescence in acidic vesicular organelles. Detection was performed through fluorescence microscopy and flow cytometry.

### Measurement of the mitochondrial function

For the live cell metabolic assay, an XF24 Analyzer (Seahorse Bioscience; Agilent Technologies, Santa Clara CA, USA) was utilized. Cells were seeded at a density of 2 × 10^4^ cells/well into their specialized 24-well microplates (Seahorse XFe24 FluxPak, Part #102340–001, Agilent Technologies). The cartridge sensor was soaked using XF Calibrant in a CO_**2**_**-free** incubator at 37 °C overnight. The culture medium was replaced by NaHCO_3_-free DMEM, followed by calibration for respiratory rate. Basal OCR levels were first measured followed by measurements of the other parameters after stimulation with oligomycin (10 μM) for ATP production and proton leak; carbonyl cyanide-4-(trifluoromethoxy) phenylhydrazone (2 μM) for maximal respiration and spare respiratory capacity; a combination of rotenone and antimycin A (5 μM) for nonmitochondrial respiration. Three measurements were recorded in each well.

To study membrane potential (ΔΨm), a ΔΨm-dependent dye, JC-1 (Invitrogen, Waltham, MA, USA) was used. This allowed cells to generate signals of red and green fluorescence from the aggregated form at high ΔΨm and the monomeric form at low ΔΨm, respectively. Detection was performed using flow cytometry.

### Chromatin immunoprecipitation (ChIP)

The study was performed with an EZ-Magna Chip A/G kit (Merck Millipore). Proteins were cross-linked to DNA with the addition of 1% formaldehyde for 10 min and then quenched by 125 mM glycine. Cells were treated with protease inhibitors and lysed in the lysis buffer. The samples were then incubated with the antibody and magnetic beads at 4 °C overnight. After magnetic separator, they were incubated at 62 °C with proteinase K. DNA was released by reverse cross-linking and was purified. Subsequent qPCR was performed using SH3GLB1 forward and reverse primers as follows: 5′-CAAGCATACAGAGGCGCCGAG-3′ and 5′-CAAGAATGGGTCAGTCGGCTC-3′, respectively.

### Tumor xenograft mouse model

The studies were approved and processed with advice from the animal ethics board. U87MG-R-luciferase-EGFP cells with or without shSH3GLB1 were used to create a xenograft model in 6–7-week-old male NOD-SCID mice. Cells were injected into the subcutaneous flank area or brain for inoculation at numbers of 2 × 10^6^ or 1 × 10^5^, respectively. Ten days later, the animals were randomly assigned for treatment. The Luciferase activity was measured by using an IVIS imaging system (Xenogen, Alameda, CA, USA) after intraperitoneal injection of firefly substrate (80 mg/kg; Promega, Madison, WI, USA). Mice were euthanized if severe neurological or intolerable physical signs appeared. The tumor tissue was extracted for subsequent experiments. The intracranial experiments were conducted according to previous studies [5] to do survival analysis.

### Statistics

Data were statistically analyzed using Prism (version 7.02, GraphPad, La Jolla, CA, USA). Differences in continuous variables were calculated using unpaired, two-tailed Student’s t-test. The linear correlation between two variables was measured by using Person’s correlation coefficient (r). Statistical significance was set at *p* < 0.05.

## Results

### Recurrent GBM shows enhanced SH3GLB1 and OXPHOS gene signatures

**Fig. 1:**
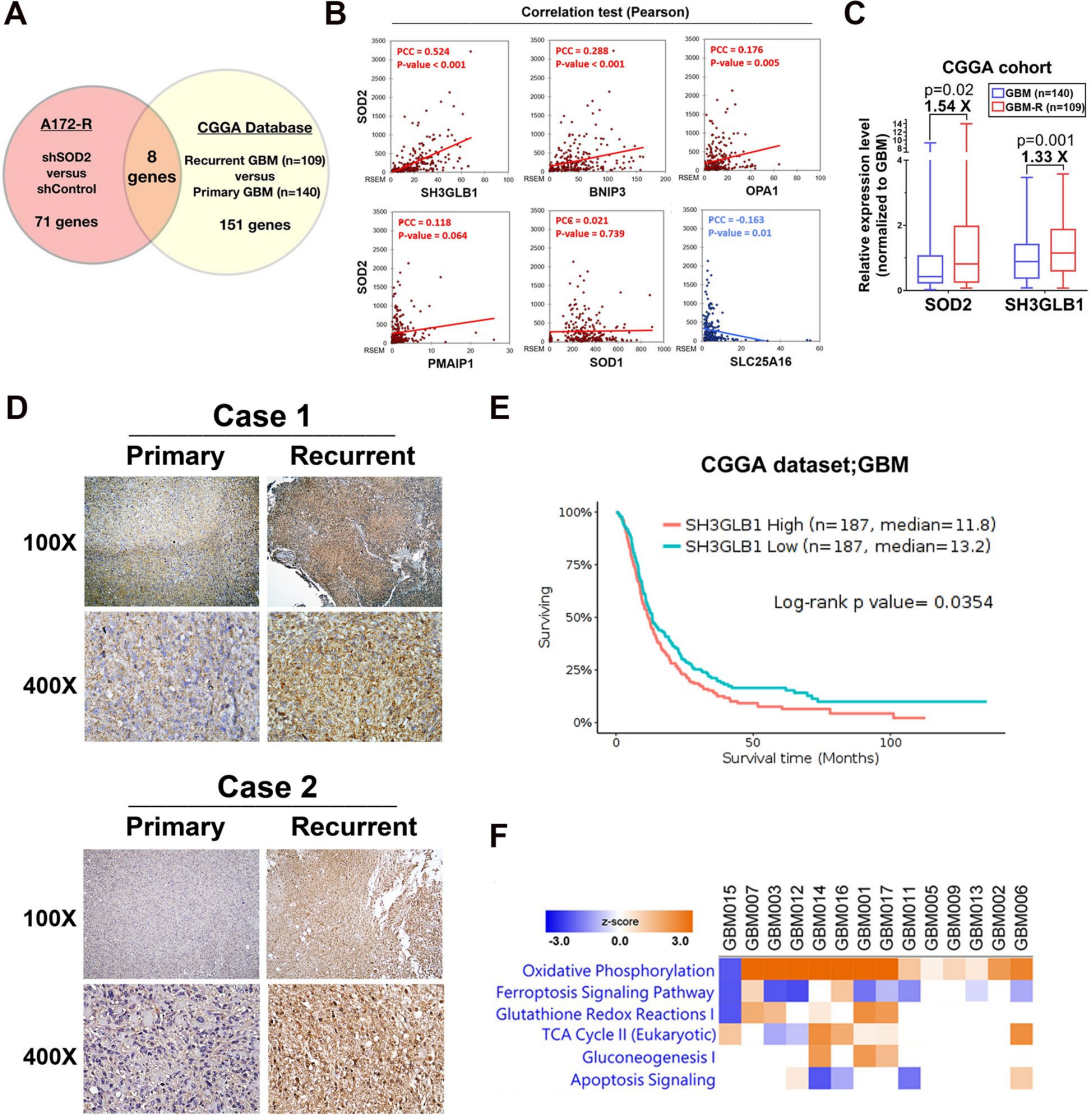
SH3GLB1 and OXPHOS-related genes are associated with recurrent GBM. **A** Eight altered mitochondria-related genes were revealed to have similar and significant trend by crossing data from the PCR Array and the CGGA database. **B** Their corresponding expression with *SOD2* in GBM cohort of the CGGA database is illustrated in scatter plots in the left panel (*SLC25A5* is not illustrated, *p* = 0.310). The levels of *SOD2* and *SH3GLB1* according to recurrent or primary GBM were shown in box plot in the right panel with statistical evaluation. **C** Expression of SH3GLB1 in the paired primary- and recurrent-tissues was revealed using IHC staining. Two in nine cases are represented here with the remaining shown in Supplementary Fig. S1C (**D**) The Kaplan–Meier graph of the *SH3GLB1* gene expression that was higher or lower than the median level in the GBM dataset from the CGGA database. **E** Ingenuity pathway analysis results of the 14 paired recurrent versus treatment-naïve glioma RNA-seq dataset. The heatmap showed selected pathways that are related to mitochondria-based functions in the “Molecular and Cellular Functions” category. Note that two grade III diseases and two having IDH1 mutations were among the samples

SOD2-knockdown A172-R cells were used to find the potential mediating genes that were associated with SOD2 for metabolic alternation (Supplementary Fig. S1A). The result was cross-examined with profiles obtained from the CGGA database that included 140 primary and 109 recurrent GBM tumors (Fig. 1A). Eight genes that could be significant in resistance-related mitochondrial functions and tumor recurrence were analyzed. Among them, SH3GLB1 exhibited a higher correlation with SOD2 (Pearson’s *r* = 0.524, *p* < 0.001, Fig. 1B left panel). The two genes also showed significant correlation in the TCGA database and in our samples (Supplementary Fig. S1B).

The CGGA database suggested higher *SH3GLB1* expression in recurrent data (Fig. 1B right panel). Supportively, we found the protein expression enhanced in eight of nine recurrent tumor tissues than in primary ones (Fig. 1C and Supplementary Fig. S1C). In both the CGGA and the TCGA databases, survival curves of cases with higher *SH3GLB1* expression showed worse prognosis (Fig. 1D and Supplementary Fig. S1D). Given that SH3GLB1 can alter mitochondrial functions [18], IPA for the RNA transcriptome of paired recurrent and treatment-naïve high-grade glioma samples was used to pinpoint related pathways. Among major mitochondria-related functional pathways, the OXPHOS pathway was predicted to similarly activate in thirteen of fourteen recurrent tumors (Fig. 1E). We noticed increased levels of multiple individual genes particularly in complexes I, III, IV, Fe/S cluster, and was supported by the CGGA database (Supplementary Fig. S2).

### SH3GLB1 is associated with tumor-initiating features in tumors

**Fig. 2:**
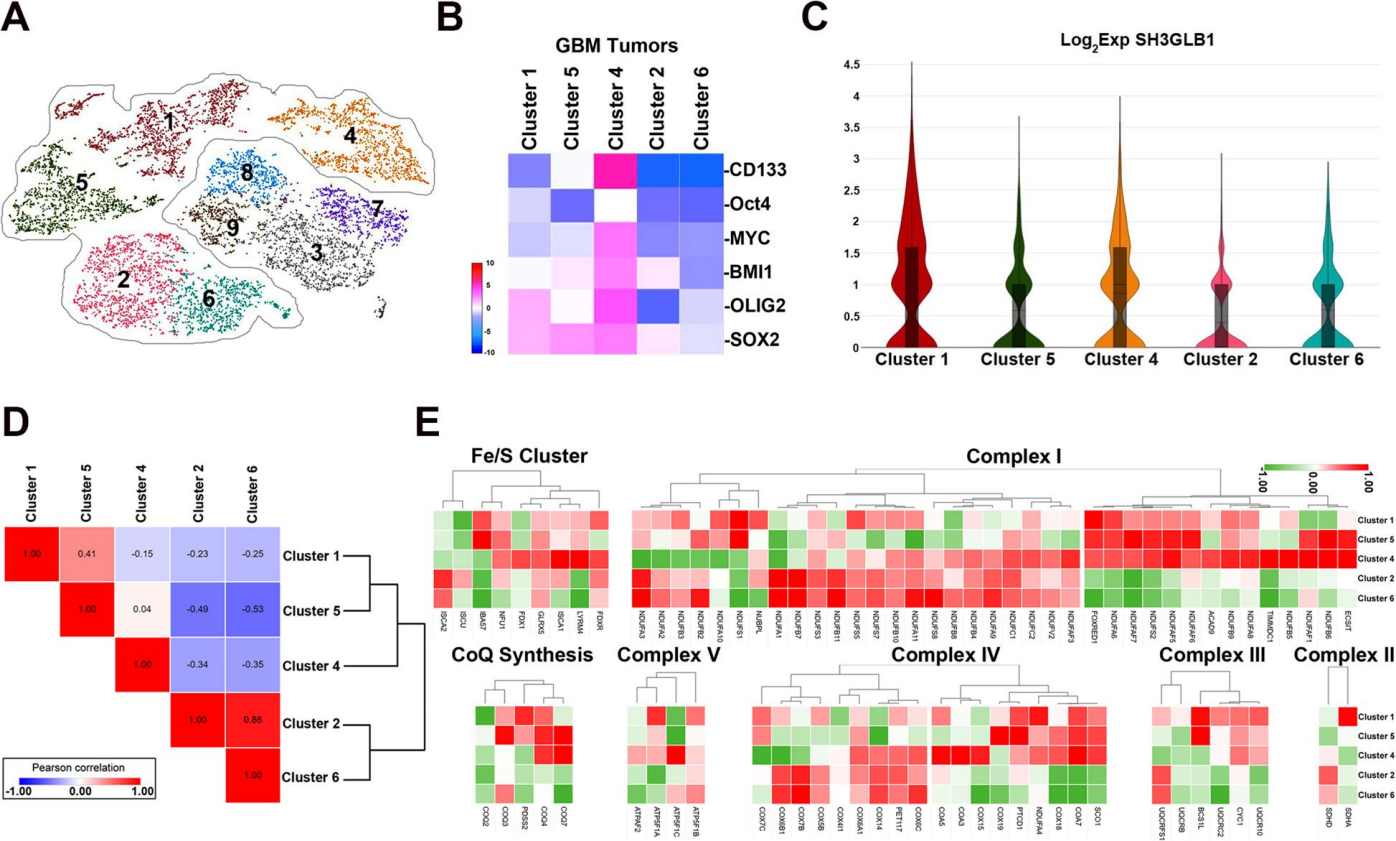
Higher SH3GLB1 levels are related to specific subsets of the tumor. **A** The tSNE plot of the scRNA transcriptome from five GBM tumor samples sorted into tumor clusters. **B** Common genes related to tumor-initiating cell features are shown in the heatmap plot. **C** Expression level of SH3GLB1 in tumor clusters is shown in the violin plot. **D** The statistical similarity matrix between the indicated clusters was based on the OXPHOS-related genes. **E** Levels of OXPHOS-related genes in the tumor clusters are shown in the heatmap by complexes

We performed scRNA transcriptomic analysis on five GBM tumors to study the intratumoral cell distribution. Among the sorted clusters based on their differences in gene expression, five were identified as tumors (number 1, 2, 4, 5, and 6; Fig. 2A and Supplementary Fig. S3). We observed that cluster 4 exhibited the most abundant markers for TICs, such as *CD133, Olig2*, *SOX2*, *Bmi1*, and *Myc* (Fig. 2B) [25]. Notably, clusters 1 and 4 exhibited significantly higher expressions of *SH3GLB1* than clusters 2 and 6 (Fig. 2C), in which both possessed the lowest levels of TIC profiles. Next, hierarchical clustering by OXPHOS genes suggested that cluster 4 expressed distinct features from clusters 2 and 6, with clusters 1 and 5 being the intermediate types (Fig. 2D). Higher expression of genes was most commonly noted in cluster 4 in the entire complexes or in the individual complexes of I, IV, and the Fe/S clusters of OXPHOS (Fig. 2E). Additionally, the genes in complex III were generally expressed higher in cluster 1. These results demonstrated that SH3GLB1 and the altered OXPHOS genes were associated with TIC-feature cells, which could inherit resistance characteristics [5].

### Sp1 in the resistant cells activates SH3GLB1 transcription

**Fig. 3:**
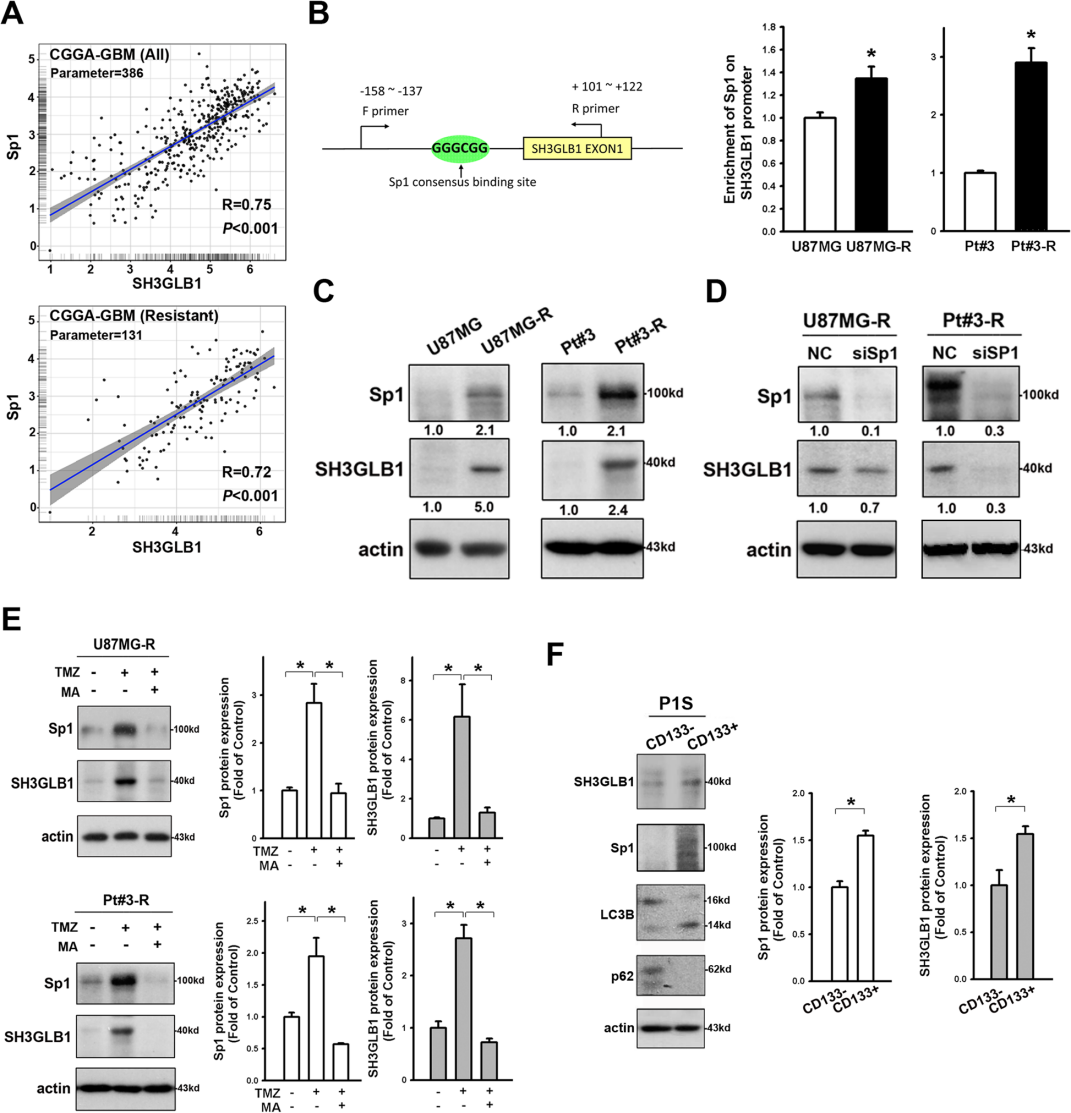
Sp1 promotes SH3GLB1 expression. **A** Genetic expression of SH3GLB1 and Sp1 from the GBM dataset of the CGGA database is shown in scatter plots with correlation assessment. **B** Schematic graph suggesting the potential Sp1 binding site on the SH3GLB1 promoter. Enhanced binding of Sp1 to the SH3GLB1 promoter is shown by chromatin immunoprecipitation assay in the resistant cells. **C** Western blot analysis showing enhanced Sp1 and SH3GLB1 expression in the resistant cells. **D** Western blot analysis showing reduced SH3GLB1 expression in the resistant cells with siSp1. **E** Western blot analysis showing that mithramycin A (MA) alleviated the TMZ-induced enhancement of Sp1 and SH3GLB1. **F** Sorted CD133^+^ and CD133^−^ subsets from patient-derived primary GBM cells. The former exhibit higher Sp1, LC3B-II and lower p62 in the Western blot analysis. *N* = 3 in each group, *p < 0.05

We previously demonstrated that upregulation of Sp1 plays crucial roles in the development of TMZ resistance in GBM [20]. By examining the data available from the CGGA database, a positive correlation between the expression of SH3GLB1 and Sp1 was found in GBM (Fig. 3A). A conserved binding site for Sp1 is present in the promoter region of endophilin family members [26]. We hereby studied TMZ-resistant GBM cells and found increased level of Sp1 binding on the promoter region of *SH3GLB1*, as compared to the drug-sensitive counterparts (Fig. 3B). The drug resistant cells also exhibited higher expression of Sp1 and SH3GLB1 comparing to the drug sensitive cells (Fig. 3C). Downregulation of Sp1 by siRNA decreased the expression of SH3GLB1 in U87MG-R and Pt#3-R cells (Fig. 3D). Inhibiting Sp1 by small molecule inhibitor, Mithramycin A, attenuated the upregulation effects of TMZ on SH3GLB1 expression (Fig. 3E). We observed higher SH3GLB1 and SP1 expression in CD133^+^ P1S cells than in their CD133^−^ counterparts (Fig. 3F) [5].

### SH3GLB1 is associated with autophagy in response to TMZ in resistant cells

**Fig. 4:**
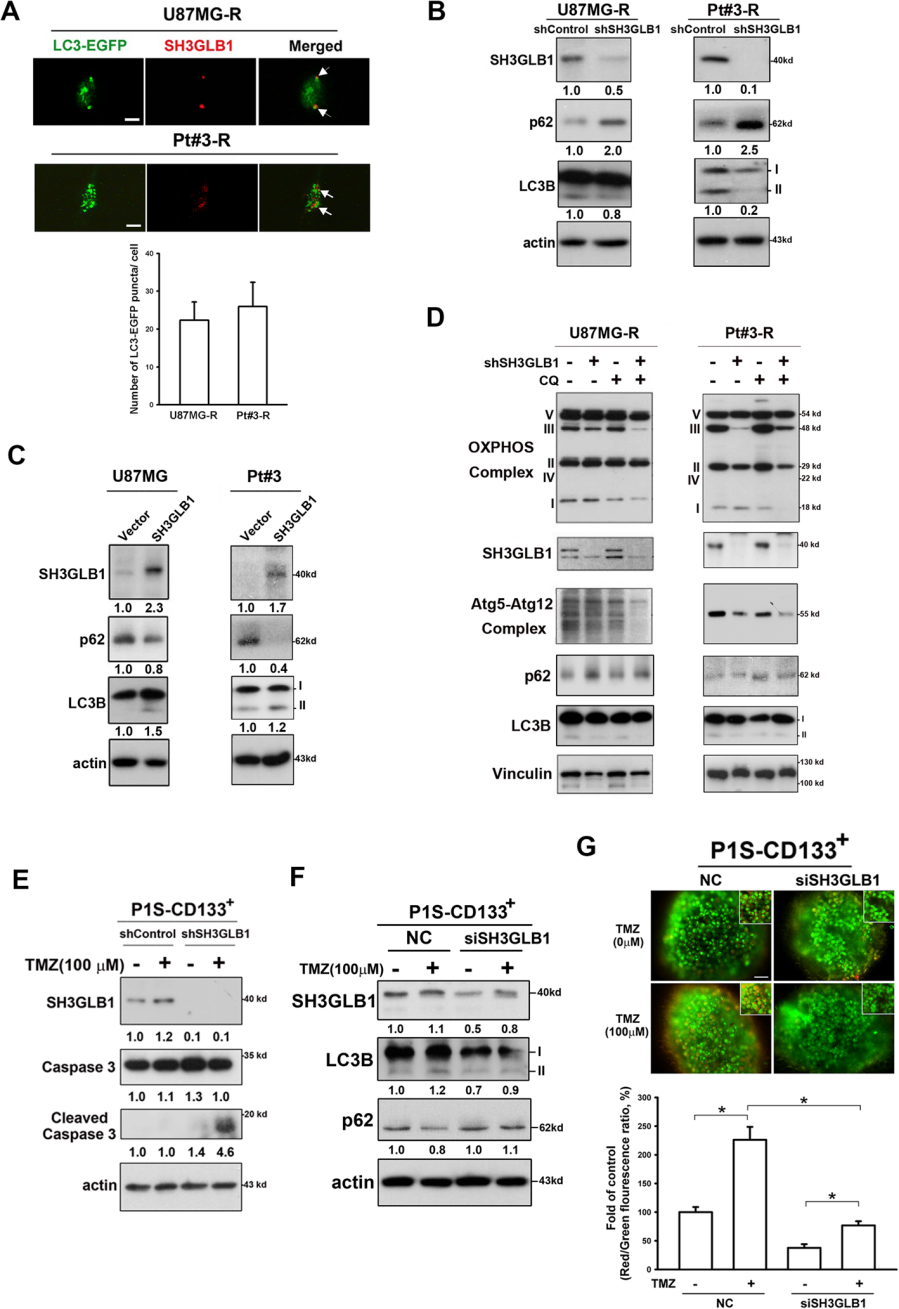
SH3GLB1 downregulation reduces TMZ-enhanced autophagy. **A** The SH3GLB1 protein (Red) is colocalized (white arrow) with autophagosomes labeled by LC3-EGFP (Green). The statistic graph showing the numbers of autophagic puncta (dot). Scale bar = 50 μm (**B**) Resistant cells treated with shSH3GLB1 exhibits attenuated autophagy levels. **C** The parental cells with SH3GLB1 overexpression exhibit enhanced autophagy levels. **D** Western blot analysis showing the expression of LC3, p62, Atg5–12 complex, and OXPHOS complexes from cells with or without SH3GLB1 knockdown that were cotreated with chloroquine (CQ). **E** Western blot analysis showing increased levels of cleaved caspase 3 in recurrent GBM spheroid cultures with siSH3GLB1 and TMZ cotreatment for 24 hours. **F** Autophagy enhanced by evidence of altered LC3B-II and p62 expression after TMZ treatment for 24 hours was attenuated in siSH3GLB1 cells. **G** Attenuated detection of induced acridine orange–stained acidic vesicular organelles is shown following TMZ treatment (24 hours) and siSH3GLB1 knockdown. The statistical graph obtained by flow cytometry is shown in the right panel (Scale bar = 200 μm; *p < 0.05; *N* = 3 in each group; R: resistance)

Autophagy has been learned to contribute to chemotherapy-resistance that is related to TIC-feature cancer cells [27]. Notably, increased LC3B-II and decreased p62 were revealed in the CD133^+^ P1S cells (Fig. 3F). To demonstrate SH3GLB1 to be crucial for autophagy in resistant cells, we first found SH3GLB1 co-localized with LC3, suggesting that its association with autophagosomes (Fig. 4A). Next, application of shSH3GLB1 in the resistant cells resulted in increased p62 expression and reduced LC3B-II expression (Fig. 4B). Conversely, SH3GLB1 overexpression in the parental cells decreased p62 expression and increased LC3B-II expression (Fig. 4C).

The functional relevance of autophagy was next investigated. Dual blockade of autophagy with shSH3GLB1 and chloroquine attenuated the Atg5–12 conjugate expression and OXPHOS in complexes I and III (Fig. 4D). ShSH3GLB1 significantly promoted caspase 3 cleavage (activation) in response to TMZ treatment in CD133^+^ P1S, suggesting that its role is related to TIC features (Fig. 4E). Likewise, SH3GLB1 knockdown in the CD133^+^ cells attenuated TMZ-induced autophagy (Fig. 4F). This also resulted in reduced formation of acidic vesicular organelles (Fig. 4G). Together, these findings revealed that SH3GLB1-related autophagy plays important roles in resistance cells.

### SH3GLB1 affects mitochondrial function and TMZ susceptibility

**Fig. 5:**
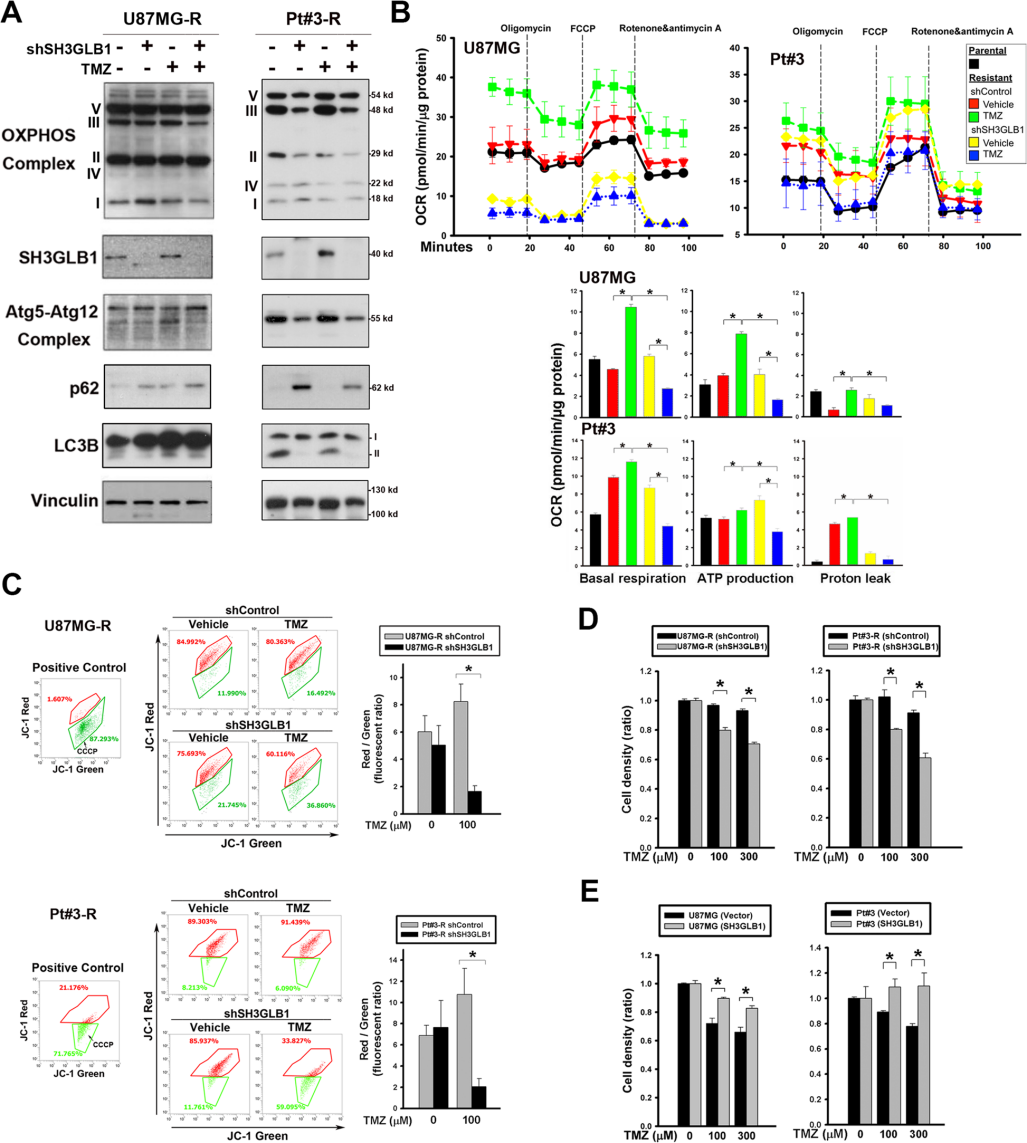
SH3GLB1 enhances mitochondrial functions and TMZ resistance. **A** Western blot analysis showing the expression of LC3, p62, Atg5–12 complex, and OXPHOS complexes from cells with or without shSH3GLB1 that were treated with TMZ. **B** OCR was measured using a Seahorse Analyzer. The individual OCR parameters were measured using the indicated reagents (left). The summary graphs of mitochondrial respiration are illustrated at right and in the Supplementary Fig. S4B. **C** Altered dynamics of ΔΨm were detected using JC-1 dye in U87MG-R cells treated with TMZ (24 hours) and shSH3GLB1. The positive control with carbonyl cyanide m-chlorophenyl hydrazone (CCCP, 10 μM for 24 hours) indicates ΔΨm impairment. The results are illustrated in the bar graph. (**D** and E) The results of cell density assays are illustrated with (**D**) control (black) or shSH3GLB1 (gray); (**E**) vector (black) or SH3GLB1-overexpressing plasmid (gray) that were treated with TMZ for 72 hours. *N* = 3 in each group, *p < 0.05

As above mentioned, SH3GLB1-induced autophagy affects assembly of OXPHOS complexes (Fig. 4D). Downregulation of SH3GLB1 by shRNA attenuated TMZ-enhanced expression of Atg5–12 conjugate and the levels of assembly in OXPHOS complexes I and III (Fig. 5A). Using a live-cell metabolic assay, a switch of metabolic phenotype (OCR/ECAR) was observed in U87MG-R and Pt#3-R cells with SH3GLB1 downregulation (Supplementary Fig. S4A). Notably, SH3GLB1 suppression alleviated TMZ-enhanced mitochondrial respiration, such as basal respiration, ATP production, and proton leakage, suggesting that SH3GLB1 regulates OXPHOS and mitochondrial activity in TMZ-resistant cells (Fig. 5B). The pattern was similarly found in respect of the maximal and the nonmitochondrial respirations but not the spare respiration capacity (Supplementary Fig. S4B). In addition, SH3GLB1 downregulation alleviated TMZ-enhanced ΔΨm, suggesting the protein is a determinant factor for mitochondria polarization in the resistant cells (Fig. 5C and Supplementary Fig. S4C).

Inhibition of cell growth was also observed when TMZ was administered to shSH3GLB1 resistant cells (Fig. 5D). Conversely, SH3GLB1 overexpression attenuated the effect of TMZ on U87MG and Pt#3 cells (Fig. 5E). The inhibitory effect of a complex 1 inhibitor, IACS-010759, was reversed by SH3GLB1 overexpression (Supplementary Fig. S5A). Similarly, TMZ-resistant cells also exhibited decreased susceptibility to IACS-010759, which was reversed by SH3GLB1 knockdown (Supplementary Fig. S5B). These results suggested that SH3GLB1 and the related OXPHOS play an important role in the induction of TMZ resistance in GBM cells.

### Downregulation of SH3GLB1 reinstates the TMZ treatment effect in xenograft GBM models

**Fig. 6:**
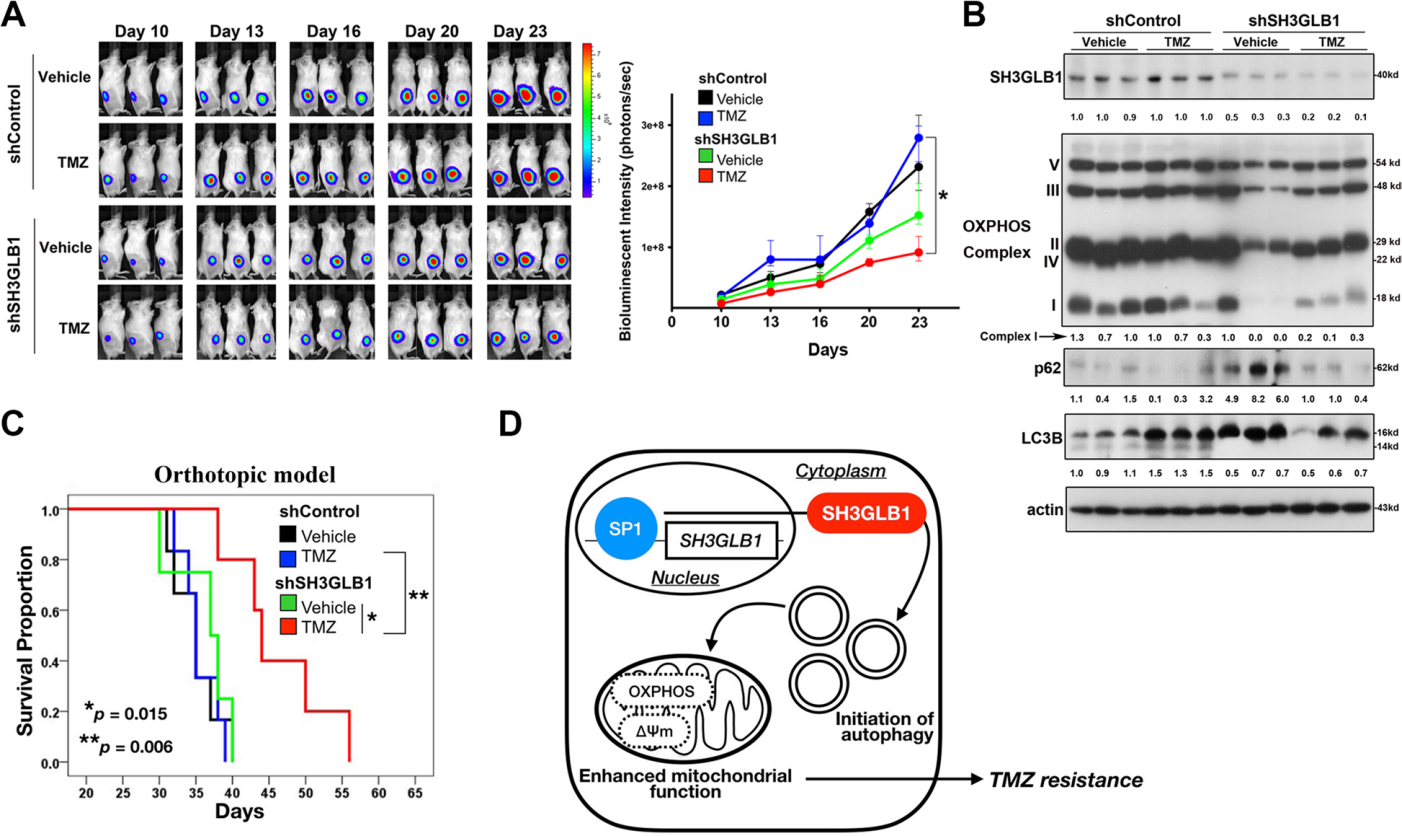
SH3GLB1-knockdown resistant cells regain treatment benefit from TMZ in the xenograft mouse model. **A** The bioluminescent signal from the luciferase-carrying U87MG-R cells in tumor carrying mice was detected using an IVIS imaging system and plotted (*N* = 6 in each group, *p < 0.05). **B** The protein immunoblot of the tumor showing SH3GLB1 and the proteins related to autophagy and OXPHOS (*N* = 3 in each group). **C** Kaplan-Meier plot showing the survival curves of the orthotopic animals in each group (*N* = 6 in each group, *p < 0.05). **D** The working scheme of SH3GLB1-regulated OXPHOS for TMZ resistance

To verify the roles of SH3GLB1 in TMZ-resistant cells in vivo, a xenograft animal model was applied with luciferase-carrying U87MG-R cells implanted into the subcutaneous area of the flank. The results revealed slower growth rate of tumors in the shSH3GLB1 group that received TMZ treatment (Fig. 6A and Supplementary Fig. S6A ~ F). Analysis of the tumors showed reduced levels of the enhanced LC3B-II in the TMZ/shSH3GLB1 group and the decreased levels of assembly in OXPHOS complex I in shSH3GLB1 groups (Fig. 6B). These findings were compatible to the results of in vitro studies. Finally, the study with the orthotopic mouse model showed prolonged survival in the shSH3GLB1 group administrated with TMZ, demonstrating the significance of SH3GLB1 in respect of disease treatment (Fig. 6C).

## Discussion

SH3GLB1 has been linked to TMZ susceptibility owing to its role in cell proliferation and invasion [28]. In this study, we confirmed its role in mitochondrial metabolism, which could lead to the induction of TMZ resistance. SH3GLB1 enhancement was associated with cells in clusters 1, 4, and 5 (Fig. 2C). These clusters expressed genetic signatures of oligodendrocyte-progenitor-like and neural-progenitor-like subsets regarding of their cellular states, differentiating them from clusters 2 and 6 (Supplementary Fig. S3B) [29]. They also showed enriched CD133, SOX2, OCT4, and OLIG2 (Fig. 2B), which were compatible to the report that these genes often biased toward both cellular states [30]. Accordingly, a higher level of SH3GLB1 and enhanced autophagy were inherited in CD133+ tumor cells (Fig. 3F) [27, 31]. Pre-existing clones with TIC-features can endure drug toxicities and cause recurrence [32]. Given the tumor-propagating capability in these two cellular states [30], the tumor-initiating potentials here can confer substantial effects in causing failure of disease control. Supportively, the increased SH3GLB1 expression in recurrent clinical samples suggested its association with the resistance (Fig. 1C). This is because TMZ has generally been applied through the National Health Insurance in Taiwan. Studies on cell lines further confirmed SH3GLB1 to be the critical factor for cell resistance against TMZ. These also excluded interference from irradiation of the tumor samples, which is often used concomitantly with the drug to treat GBM. Notably, we identified Sp1-mediated promotor regulation (Fig. 3). Accumulation of the transcriptional factor contributes to the TIC-resistant features of SOD2, thereby promoting ROS regulation [33]. Simultaneously, SH3GLB1 is enhanced for metabolic regulation, which is supported by the data here. SH3GLB1 is crucial for normal brain function, as well as aiding in recovery from injury [34]. It is therefore not surprising that it can enhance the disease progression of the brain tumor, leading to more serious clinical outcomes.

Traditionally, SH3GLB1 is known as a tumor-suppressor [35]. However, it has also been reported to affect cancer in other ways [36]. SH3GLB1 can promote cell death by inducing apoptosis through Bax, or by redirecting to necrosis through autophagy and GSK3β inhibition [37, 38]. It has been reported that downregulation of SH3GLB1 contributed to tumorigenesis by upregulating mitochondrial function in melanoma cells. However, this reaction was not associated with autophagy, and it was shown that the cells had equal sensitivity to anti-tumor treatments, which were not affected by SH3GLB1 levels [39]. Nonetheless, under certain circumstances, SH3GLB1 can enhance tumor migration and metastasis [40]. Additionally, results from the current study showed that SH3GLB1 enhances tumor resistance in TMZ conditions, with reduced activation of caspase 3. Further studies revealed that SH3GLB1 repressed the N-terminal epitope 6A7 of Bax in GBM models, suggesting the anti-apoptosis effect (data not shown). The protein-induced autophagy and reprogrammed OXPHOS in the resistant cells contributed toward resisting the anti-tumor effects of TMZ, highlighting the contribution of our study to the existing literatures.

Despite the general consensus that inhibition of autophagy process enhances the anti-tumor effect of TMZ in glioma cells [41], CQ clinical trials have not reached a clear conclusion in terms of treatment outcome [42, 43]. Autophagy exerts various pro- and anti-tumor effects, therefore, it is important to dissect and study the impacts in detail to better understand the complexity of this phenomenon. The scRNA transcriptome of clinical samples revealed that SH3GLB1 was most prevalent in cells with the most altered OXPHOS genes (Fig. 2). On a cellular level, a decrease in autophagy by CQ and shSH3GLB1 resulted in reduced expression of OXPHOS genes (Fig. 4D). The mechanism of SH3GLB1-autophagy in regulating cell metabolism was not elucidated here. However, it has been reported that the fatty acid production catabolized by autophagy mediated the OXPHOS reaction [10]. We then determined that SH3GLB1-related autophagy is functionally associated with the development of TMZ resistance, by regulating OXPHOS. This warrants exploration of potential anti-autophagy strategies to restore the anti-tumor effects of TMZ as well as a detailed investigation of the underlying metabolism.

Expression of individual OXPHOS genes varied in these clinical data as well as in the CGGA database (Supplementary Fig. S2). Genes encoding the core subunits of the complexes can affect the malignant cells in opposing manners. For example, low *NDUFS1* and high *NDUFS8* expression levels in lung cancer were found to predict poor overall survival [44]. In our study, higher levels of NDUFAF7 (complex I) and ATP5F1A (complex V) were related to more severe clinical prognoses, and were clearly enhanced in TIC-feature clusters (Fig. 2E and Supplementary Fig. S7A–B). However, more positive clinical prognoses were observed when NDUFB10 (complex I), NDUFB2 (complex I), SDHA (complex II), UQCRFS1 (complex III), UQCRB (complex III), COX7C (complex IV), IBA57 (Fe/S clusters), and CoQ3 (CoQ synthesis) were higher (Supplementary Fig. S7C-J), but had no tendency related to TIC-features in our data (Fig. 2E). Alterations in single genes can be functionally redundant and cause discrepancies, leading to incompatible results. Therefore, a functional assessment related to mitochondrial complexes would be more representative [45]. We found that SH3GLB1 promoted metabolism during TMZ treatment by altering ΔΨm and mitochondrial respiration (Fig. 5). Notably, SH3GLB1 did not consistently affect ECAR (Supplementary Fig. S4A), suggesting that resistant cells are more prone to utilize OXPHOS than glycolysis [9]. In summary, the network is functionally important for TIC features in TMZ-resistant cells [46]. Therefore, the OXPHOS profiles for the resilient tumor subsets provide important information.

Alteration of metabolic profiles has been suggested as a potential strategy for cancer treatment. With the discovery of high-affinity complex I inhibitors, major advances in research is expected by uncovering the benefits of suppressing intracellular OXPHOS function. For example, potential synergistic therapy with other anticancer agents has been suggested in a breast cancer model [47]. Selection of suitable candidates is vital because modulating metabolism causes varying results [48]. For example, treatment resistance in tumors can depend on the deregulated metabolic reprogramming [49]. Additionally, SH3GLB1 levels can potentially serve as a GBM cell indicator as they antagonized the complex I inhibitor effect (Supplementary Fig. S5). In the resistant cells, shSH3GLB1 attenuated TMZ-enhanced OXPHOS significantly by blocking the assembly of complex 1 (Figs. 5A and 6B). Our findings warrant further exploration for strategies to maximize the effect of OXPHOS-related therapies.

## Conclusions

We found that SH3GLB1 enrichment in TIC-feature subsets enhances autophagy, leading to increased ΔΨm and enhanced OXPHOS (Fig. 6D). The importance of SH3GLB1 in the promotion of TMZ resistance was also verified using animal experiments and clinical data. SH3GLB1 was regulated via Sp1, and together they contribute to cell evasion from TMZ cytotoxicity in GBM. It is noteworthy that the data could be replicated in other cell lines including A172 and pt.#5 (Supplementary Figs. S8 and S9), confirming the impact of the protein in cell metabolism. However, this study does not exclude the possibility that SH3GLB1 regulates the OXPHOS genes by other means. Nevertheless, valuable contributions in terms of SH3GLB1 have been highlighted. This deserves further attention regarding the effect of TMZ or novel OXPHOS modulation strategies in future studies.

## Supplementary Information

**Additional file 1: Figure S1.** (A) Differentiation of mitochondria-related genes in A172 TMZ resistance cells (A172-R) with shSOD2 or shControl is shown in the heatmap graphs. A total of 84 genes was assessible from the assay. Among them, however, only 71 of the items were found in the database of mitochondria-related genes according to MitoCarta 2.0. (B) The correlation between SH3GLB1 and SOD2 was also shown in TCGA-GBM dataset and the 14 paired patients. (C) Nine cases showing expression of SH3GLB1 in the paired primary- and recurrent-tissues using IHC staining (100x and 400x magnification). (D) Kaplan-Meier curves of TCGA-GBMLGG (GBM and low-grade glioma) database showed higher SH3GLB1 levels caused poor survival. **Figure S2.** Ingenuity Pathway Analysis was applied for analysis of the major mitochondria-related molecular and cellular functional alterations in the transcriptome data from 14 RNA-seq data from paired recurrent and treatment-naïve high-grade glioma samples. As shown in the figure, sample numbers of down- (left column) or up-regulated (right column) genes. In each grid, the color is determined by the rank percentile, and the number represents sample that fulfills the criteria. The levels and significance of the genes ratio from CGGA glioma database (recurrent versus primary) are also shown aside. Note that none of the genes in complex II had CGGA database significance better than **, and the genes in complex V and CoQ synthesis was not higher in the resistant groups in the 14 paired samples. ***p < 0.001, **p < 0.01, *p < 0.05, NS: not significant. NA: data not available. **Figure S3.** (A) Five naïve glioblastoma tumor samples were used for single-cell transcriptome and were sorted into nine clusters according to their gene expression (Fig. 2A). The heatmap graph shows the common related genes of GBM and tumor-initiating cells among the clusters. Note that clusters 3, 7, 8, and 9 are identified as immunocytes because of the presence of the markers such as CD3E/G, GZMA, ITGAM, ITGAX. (B) The clusters reclassified by the studies of Neftel et al. (Cell, 2019) were divided into four cellular states. **Figure S4.** (A) Cell metabolic phenotype profile were measured using Seahorse Analyzer (OCR and ECAR). (B) The individual OCR parameters were measured using the indicated reagents and plotted in Fig. 5B, with the statistic graphs showing the parameters of maximal respiration, spare respiratory capacity, and nonmitochondrial respiration. (C) The parental cells transfected with SH3GLB1-overexpressing vector were stained with a JC-1 dye, showing the enhanced fluorescent ratio. **Figure S5.** (A) IACS-010759 effect to cell density for 72 hours of the parental cells with or without SH3GLB1 overexpression are shown in the bar graph. (B) IACS-010759 effect to cell density for 72 hours of the resistant cells with or without SH3GLB1 knockdown are shown in the bar graph. (*P < 0.05) **Figure S6.** (A) The U87MG-R luciferase-carrying cells (U87MG-R-luc-EGFP) were transfected with shSH3GLB1 used in animal experiment. SOD2 levels did not be affected by SH3GLB1 deficiency. (B) The tumor volume was measured at Day 20 and Day 23 according to the National Cancer Institute formula. (C) The body weight was recorded at the indicated day as the statistic graph. (*P < 0.05) (D) The image shows the tumor extracted from euthanized mice (scale bar = 1 cm) (E) The SH3GLB1 expression in the tissue using an IHC staining is shown (scale bar = 200 μm). (F) The body weight was recorded and shown in the statistical graph. **Figure S7.** Kaplan-Meier curves of GBM database from CGGA showed the survival of the indicated subunits genes of OXPHOS complex. **Figure S8.** A172 or A172-R cells are used in the results including roles of SH3GLB1 on Sp1 promoter, Sp1 expression, 36 autophagy levels, OXPHOS levels, cell density and OCR analysis. **Figure S9.** Pt#5 or Pt#5-R cells are used in the results including roles of SH3GLB1 on Sp1 promoter, Sp1 expression, autophagy levels, OXPHOS levels, cell density and OCR analysis.

## Data Availability

All data found in the present study are available within the article and supplementary data.

## Abbreviations

***CCCP***: Carbonyl cyanide m-chlorophenyl hydrazone
CGGA: Chinese Glioma Genome Atlas
ChIP: Chromatin immunoprecipitation
CQ: Chloroquine
ΔΨm: Membrane potential
ECAR: Extracellular acidification rate
GBM: Glioblastoma
IHC: Immunohistochemistry
IPA: Ingenuity pathway analysis
OCR: Oxygen consumption rate
OXPHOS: Oxidative phosphorylation
ROS: Reactive oxygen species
RSEM: RNA-Seq by Expectation Maximization
scRNA transcriptome: Single-cell RNA transcriptome
shRNA: Short hairpin RNA
siRNA: Small interfering RNA
SOD2: Superoxide dismutase 2
Sp1: Specificity protein 1
TICs: Tumor-initiating cells
TMZ: Temozolomide
