# Correction: SH3GLB1-related autophagy mediates mitochondrial metabolism to acquire resistance against temozolomide in glioblastoma

**DOI:** 10.1186/s13046-025-03445-0

**Published:** 2025-06-27

**Authors:** Chia-Hung Chien, Wen-Bin Yang, Jian-Ying Chuang, Jung-Shun Lee, Wei-An Liao, Chih-Yuan Huang, Pin-Yuan Chen, An-Chih Wu, Shun-Tai Yang, Chien-Cheng Lai, Pei-I. Chi, Jui-Mei Chu, Siao Muk Cheng, Chan-Chuan Liu, Daw-Yang Hwang, Shang-Hung Chen, Kwang-Yu Chang

**Affiliations:** 1 National Institute of Cancer Research, National Health Research Institutes, Tainan, Taiwan; 2 School of Medicine, I-Shou University, Kaohsiung, Taiwan; 3 TMU Research Center of Neuroscience, Taipei Medical University, Taipei, Taiwan; 4 The Ph.D. Program for Neural Regenerative Medicine, College of Medical Science and Technology, Taipei Medical University, Taipei, Taiwan; 5 Department of Biomedical Science and Environmental Biology, Kaohsiung Medical University, Kaohsiung, Taiwan; 6 Division of Neurosurgery, Department of Surgery, National Cheng Kung University Hospital, College of Medicine, National Cheng Kung University, Tainan, Taiwan; 7 Department of Cell Biology and Anatomy, College of Medicine, National Cheng Kung University, Tainan, Taiwan; 8 Department of Pathology, National Cheng Kung University Hospital, College of Medicine, National Cheng Kung University, Tainan, Taiwan; 9 Department of Neurosurgery, Chang Gung Memorial Hospital at Keelung, Keelung, Taiwan; 10 School of Medicine, Chang Gung University, Taoyuan, Taiwan; 11 Department of Neurosurgery, Chang Gung Memorial Hospital at Linkou, Taoyuan, Taiwan; 12 Graduate Institute of Medical Sciences, College of Medicine, Taipei Medical University, Taipei, Taiwan; 13 Division of Neurosurgery, Shuang-Ho Hospital, Taipei Medical University, Taipei, Taiwan; 14 Department of Oncology, National Cheng Kung University Hospital, College of Medicine, National Cheng Kung University, Tainan, Taiwan

**Correction: J Exp Clin Cancer Res 41, 220 (2022)**

**https://doi.org/10.1186/s13046-022–02429-8**

Following the publication of the original article [1], the authors discovered an error in Fig. 3D and the third panel from the left in Figure S9A, where Western blot images in the Actin blots were inadvertently duplicated. This oversight was not detected by either the authors or the reviewers during the review process. The correct figures are presented below:

**Incorrect Fig.** 3

**Fig. 3:**
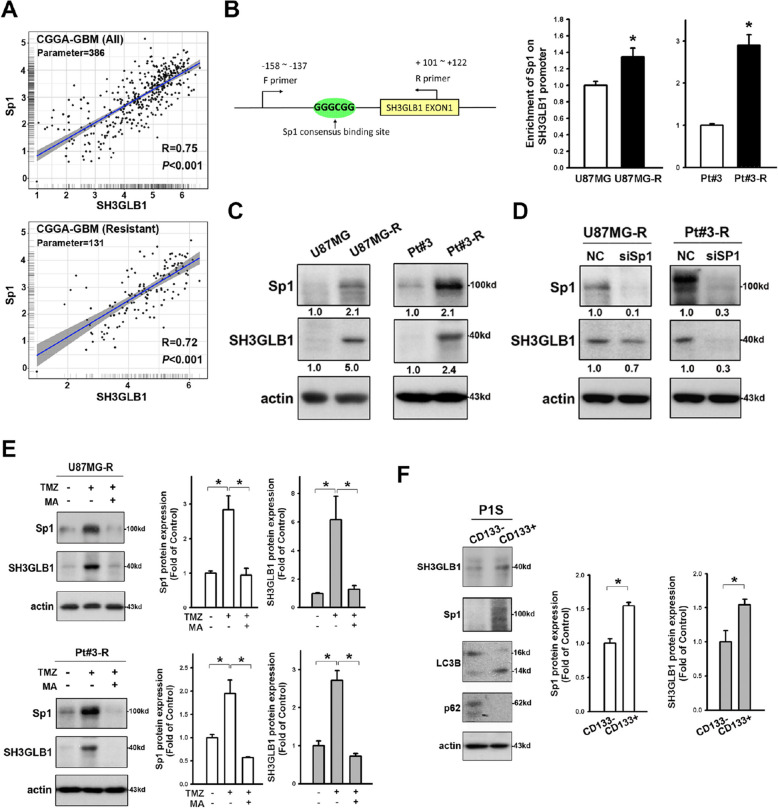
Sp1 promotes SH3GLB1 expression. **A** Genetic expression of SH3GLB1 and Sp1 from the GBM dataset of the CGGA database is shown in scatter plots with correlation assessment. **B** Schematic graph suggesting the potential Sp1 binding site on the SH3GLB1 promoter. Enhanced binding of Sp1 to the SH3GLB1 promoter is shown by chromatin immunoprecipitation assay in the resistant cells. **C** Western blot analysis showing enhanced Sp1 and SH3GLB1 expression in the resistant cells. **D** Western blot analysis showing reduced SH3GLB1 expression in the resistant cells with siSp1. **E** Western blot analysis showing that mithramycin A (MA) alleviated the TMZ-induced enhancement of Sp1 and SH3GLB1. **F** Sorted CD133^+^ and CD133.^−^ subsets from patient-derived primary GBM cells. The former exhibit higher Sp1, LC3B-II and lower p62 in the Western blot analysis. *N* = 3 in each group, *p < 0.05

**Correct Fig.** 3

**Fig. 3:**
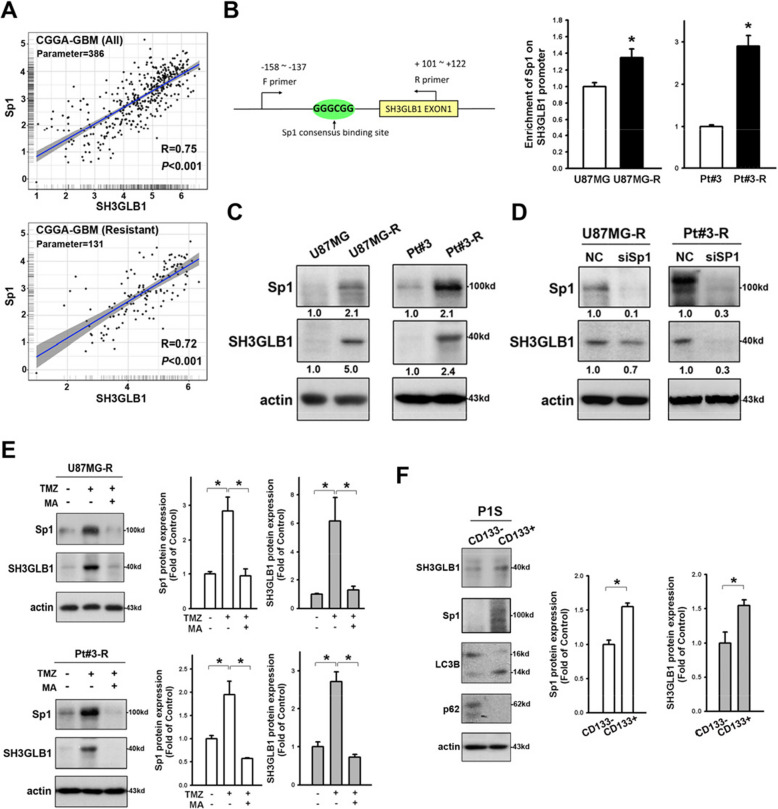
Sp1 promotes SH3GLB1 expression. **A** Genetic expression of SH3GLB1 and Sp1 from the GBM dataset of the CGGA database is shown in scatter plots with correlation assessment. **B** Schematic graph suggesting the potential Sp1 binding site on the SH3GLB1 promoter. Enhanced binding of Sp1 to the SH3GLB1 promoter is shown by chromatin immunoprecipitation assay in the resistant cells. **C** Western blot analysis showing enhanced Sp1 and SH3GLB1 expression in the resistant cells. **D** Western blot analysis showing reduced SH3GLB1 expression in the resistant cells with siSp1. **E** Western blot analysis showing that mithramycin A (MA) alleviated the TMZ-induced enhancement of Sp1 and SH3GLB1. **F** Sorted CD133^+^ and CD133.^−^ subsets from patient-derived primary GBM cells. The former exhibit higher Sp1, LC3B-II and lower p62 in the Western blot analysis. *N* = 3 in each group, *p < 0.05

**Incorrect Figure S9A**

**Figure:**
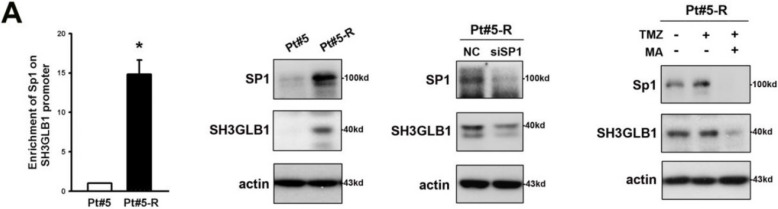
Figure S9. Pt#5 or Pt#5-R cells are used in the results including roles of SH3GLB1 on Sp1 promoter, Sp1 expression, autophagy levels, OXPHOS levels, cell density and OCR analysis

**Correct Figure S9A**

**Figure:**
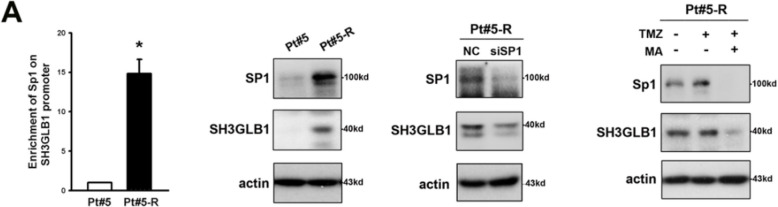
Figure S9. Pt#5 or Pt#5-R cells are used in the results including roles of SH3GLB1 on Sp1 promoter, Sp1 expression, autophagy levels, OXPHOS levels, cell density and OCR analysis

The correction does not compromise the validity of the conclusions and the overall content of the article. The original article [1] has been updated.
