# Supplementary material for: SH3GLB1-related autophagy mediates mitochondrial metabolism to acquire resistance against temozolomide in glioblastoma
Source: J Exp Clin Cancer Res. 2022 Jul 13;41:220. doi: 10.1186/s13046-022-02429-8 (PMC9281043; doi:10.1186/s13046-022-02429-8)
Supplement: Supplementary file 1 — Additional file 1: Figure S1. (A) Differentiation of mitochondria-related genes in A172 TMZ resistance cells (A172-R) with shSOD2 or shControl is shown in the heatmap graphs. A total of 84 genes was assessible from the assay. Among them, however, only 71 of the items were found in the database of mitochondria-related genes according to MitoCarta 2.0. (B) The correlation between SH3GLB1 and SOD2 was also shown in TCGA-GBM dataset and the 14 paired patients. (C) Nine cases showing expression of SH3GLB1 in the paired primary- and recurrent-tissues using IHC staining (100x and 400x magnification). (D) Kaplan-Meier curves of TCGA-GBMLGG (GBM and low-grade glioma) database showed higher SH3GLB1 levels caused poor survival. Figure S2. Ingenuity Pathway Analysis was applied for analysis of the major mitochondria-related molecular and cellular functional alterations in the transcriptome data from 14 RNA-seq data from paired recurrent and treatment-naïve high-grade glioma samples. As shown in the figure, sample numbers of down- (left column) or up-regulated (right column) genes. In each grid, the color is determined by the rank percentile, and the number represents sample that fulfills the criteria. The levels and significance of the genes ratio from CGGA glioma database (recurrent versus primary) are also shown aside. Note that none of the genes in complex II had CGGA database significance better than **, and the genes in complex V and CoQ synthesis was not higher in the resistant groups in the 14 paired samples. ***p < 0.001, **p < 0.01, *p < 0.05, NS: not significant. NA: data not available. Figure S3. (A) Five naïve glioblastoma tumor samples were used for single-cell transcriptome and were sorted into nine clusters according to their gene expression (Fig. 2A). The heatmap graph shows the common related genes of GBM and tumor-initiating cells among the clusters. Note that clusters 3, 7, 8, and 9 are identified as immunocytes because of the presence of the markers such as [file 13046_2022_2429_MOESM1_ESM.pdf]

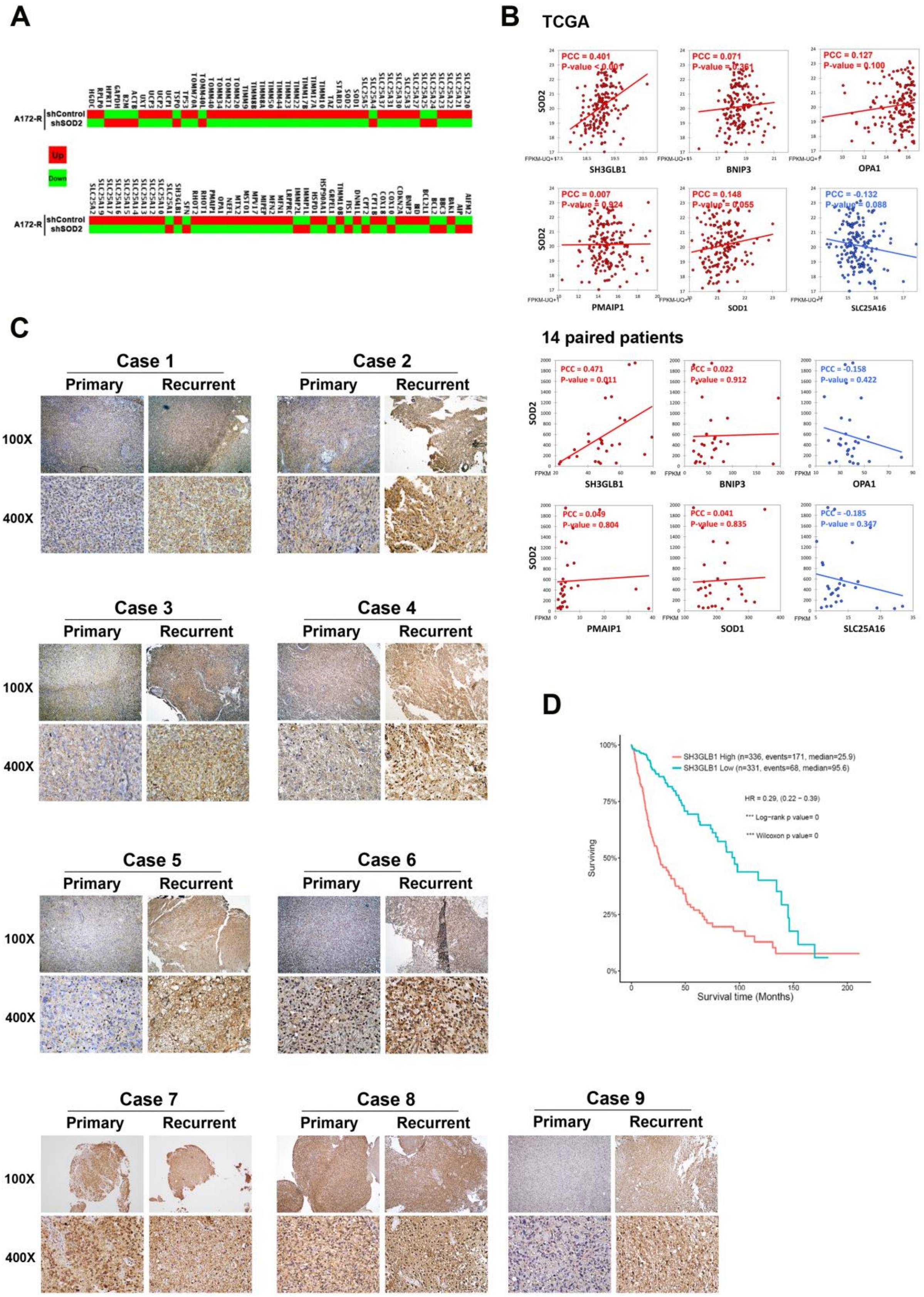
A
B
TCGA
14 paired patients
C
Case 1
Case 2
Case 3
Case 4
Case 5
Case 6
Case 7
Case 8
Case 9
Primary
Recurrent
100X
400X
D
SH3GLB1 High (n=336, events=171, median=25.9)
SH3GLB1 Low (n=331, events=68, median=95.6)
HR = 0.29, (0.22 - 0.39)
*** Log-rank p value= 0
*** Wilcoxon p value= 0
Surviving
Survival time (Months)

Figure S1

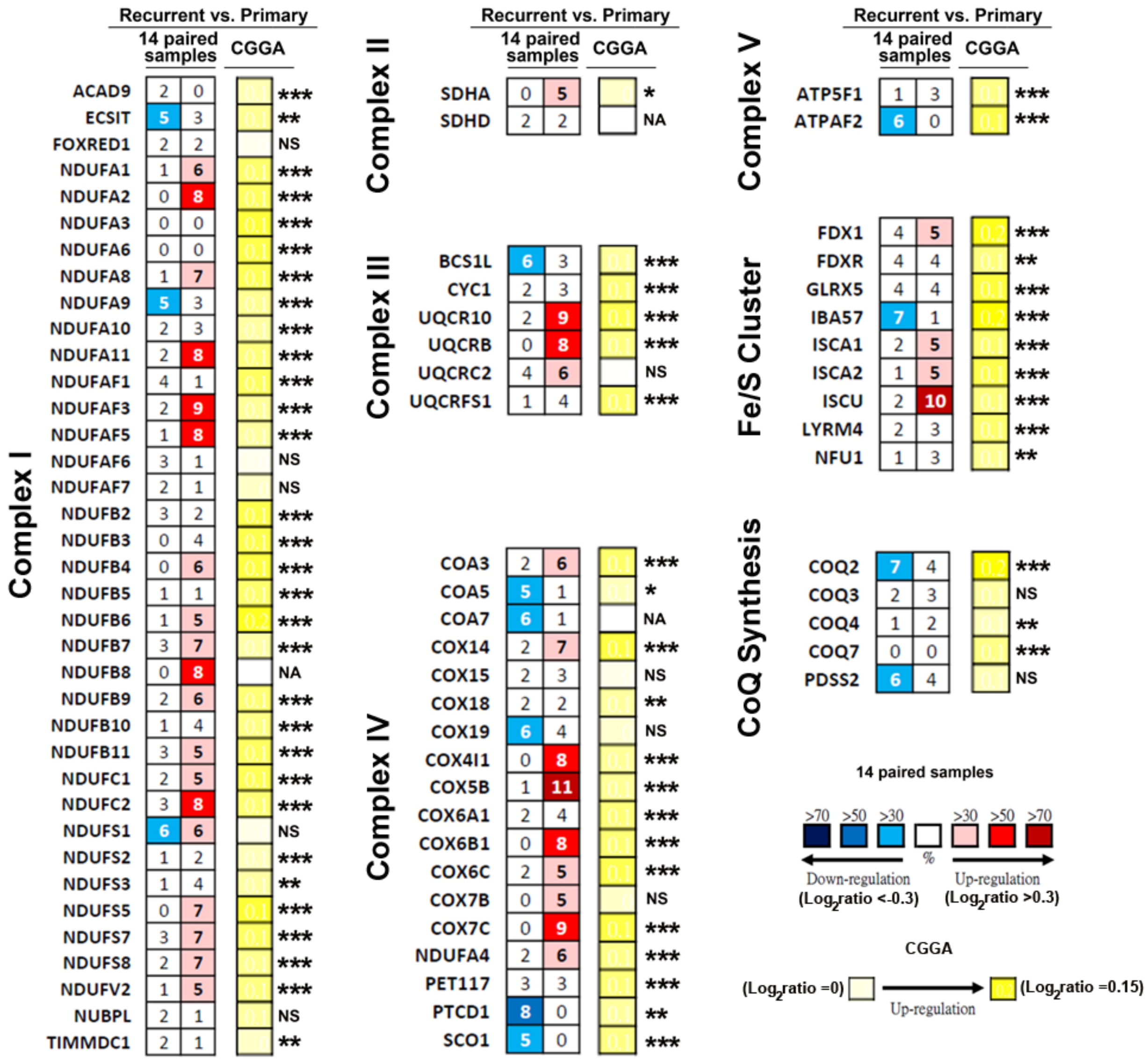
Recurrent vs. Primary
14 paired samples
CGGA
Complex I
ACAD9 2 0 ***
ECSIT 5 3 **
FOXRED1 2 2 NS
NDUFA1 1 6 ***
NDUFA2 0 8 ***
NDUFA3 0 0 ***
NDUFA6 0 0 ***
NDUFA8 1 7 ***
NDUFA9 5 3 ***
NDUFA10 2 3 ***
NDUFA11 2 8 ***
NDUFAF1 4 1 ***
NDUFAF3 2 9 ***
NDUFAF5 1 8 ***
NDUFAF6 3 1 NS
NDUFAF7 2 1 NS
NDUFB2 3 2 ***
NDUFB3 0 4 ***
NDUFB4 0 6 ***
NDUFB5 1 1 ***
NDUFB6 1 5 ***
NDUFB7 3 7 ***
NDUFB8 0 8 NA
NDUFB9 2 6 ***
NDUFB10 1 4 ***
NDUFB11 3 5 ***
NDUFC1 2 5 ***
NDUFC2 3 8 ***
NDUFS1 6 6 NS
NDUFS2 1 2 ***
NDUFS3 1 4 **
NDUFS5 0 7 ***
NDUFS7 3 7 ***
NDUFS8 2 7 ***
NDUFV2 1 5 ***
NUBPL 2 1 NS
TIMMDC1 2 1 **
Complex II
SDHA 0 5 *
SDHD 2 2 NA
Complex III
BCS1L 6 3 ***
CYC1 2 3 ***
UQCR10 2 9 ***
UQCRB 0 8 ***
UQCRC2 4 6 NS
UQCRFS1 1 4 ***
Complex IV
COA3 2 6 ***
COA5 5 1 *
COA7 6 1 NA
COX14 2 7 ***
COX15 2 3 NS
COX18 2 2 **
COX19 6 4 NS
COX4I1 0 8 ***
COX5B 1 11 ***
COX6A1 2 4 ***
COX6B1 0 8 ***
COX6C 2 5 ***
COX7B 0 5 NS
COX7C 0 9 ***
NDUFA4 2 6 ***
PET117 3 3 ***
PTCD1 8 0 **
SCO1 5 0 ***
Complex V
ATP5F1 1 3 ***
ATPAF2 6 0 ***
Fe/S Cluster
FDX1 4 5 ***
FDXR 4 4 **
GLRX5 4 4 ***
IBA57 7 1 ***
ISCA1 2 5 ***
ISCA2 1 5 ***
ISCU 2 10 ***
LYRM4 2 3 ***
NFU1 1 3 **
CoQ Synthesis
COQ2 7 4 ***
COQ3 2 3 NS
COQ4 1 2 **
COQ7 0 0 ***
PDSS2 6 4 NS
14 paired samples
>70 >50 >30 % >30 >50 >70
Down-regulation (Log2ratio <-0.3)
Up-regulation (Log2ratio >0.3)
CGGA
(Log2ratio =0) Up-regulation (Log2ratio =0.15)

Figure S2

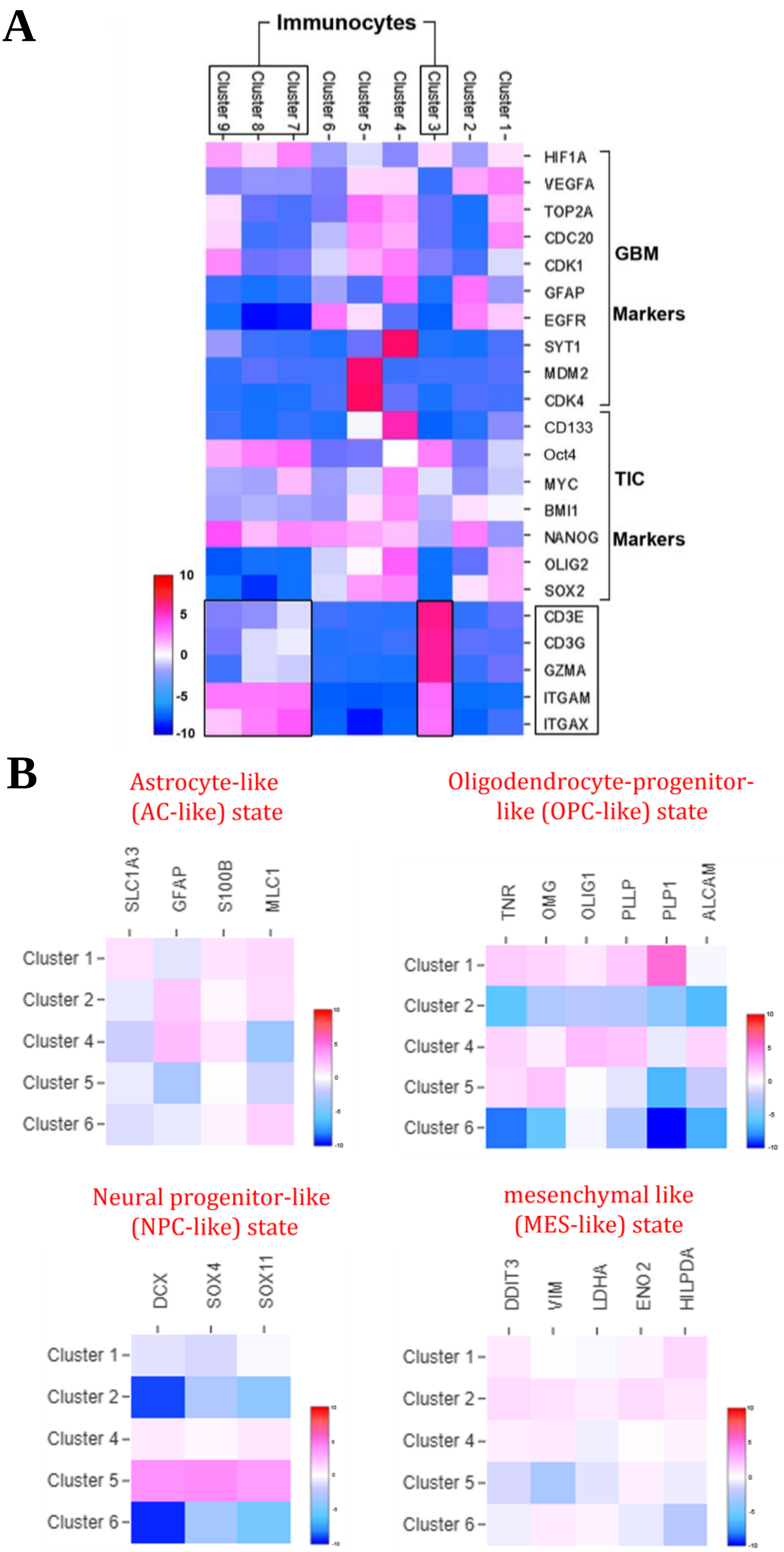
A
Immunocytes
Cluster 9
Cluster 8
Cluster 7
Cluster 6
Cluster 5
Cluster 4
Cluster 3
Cluster 2
Cluster 1
HIF1A
VEGFA
TOP2A
CDC20
CDK1
GFAP
EGFR
SYT1
MDM2
CDK4
GBM
Markers
CD133
Oct4
MYC
BMI1
NANOG
OLIG2
SOX2
TIC
Markers
CD3E
CD3G
GZMA
ITGAM
ITGAX
10
5
0
-5
-10
B
Astrocyte-like
(AC-like) state
SLC1A3
GFAP
S100B
MLC1
Cluster 1
Cluster 2
Cluster 4
Cluster 5
Cluster 6
Oligodendrocyte-progenitor-
like (OPC-like) state
TNR
OMG
OLIG1
PLLP
PLP1
ALCAM
Cluster 1
Cluster 2
Cluster 4
Cluster 5
Cluster 6
Neural progenitor-like
(NPC-like) state
DCX
SOX4
SOX11
Cluster 1
Cluster 2
Cluster 4
Cluster 5
Cluster 6
mesenchymal like
(MES-like) state
DDIT3
VIM
LDHA
ENO2
HILPDA
Cluster 1
Cluster 2
Cluster 4
Cluster 5
Cluster 6

Figure S3

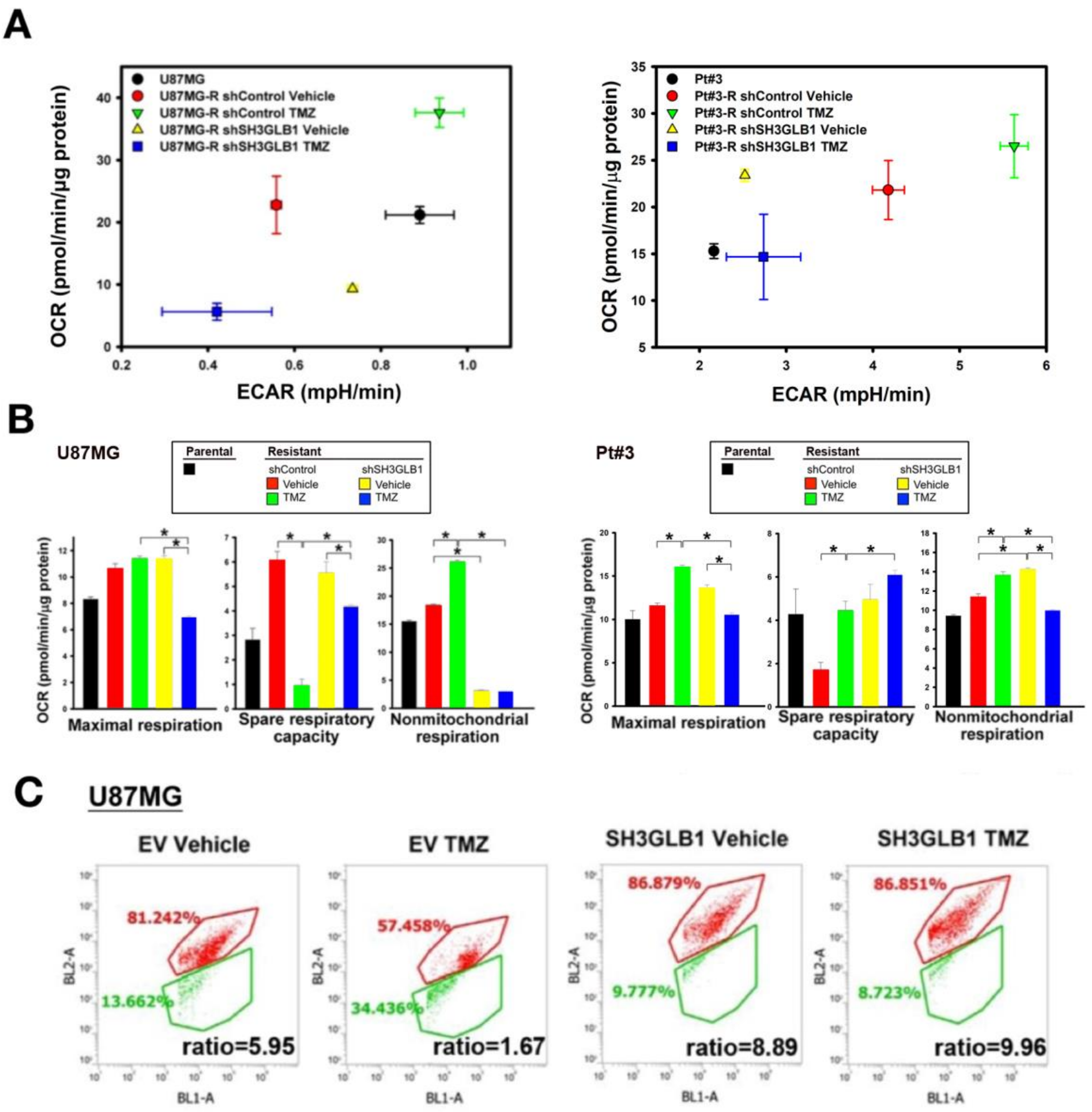
A
U87MG
U87MG-R shControl Vehicle
U87MG-R shControl TMZ
U87MG-R shSH3GLB1 Vehicle
U87MG-R shSH3GLB1 TMZ
OCR (pmol/min/µg protein)
ECAR (mph/min)
Pt#3
Pt#3-R shControl Vehicle
Pt#3-R shControl TMZ
Pt#3-R shSH3GLB1 Vehicle
Pt#3-R shSH3GLB1 TMZ
B
U87MG
Pt#3
Parental
Resistant
shControl
shSH3GLB1
Vehicle
TMZ
Maximal respiration
Spare respiratory capacity
Nonmitochondrial respiration
C
U87MG
EV Vehicle
EV TMZ
SH3GLB1 Vehicle
SH3GLB1 TMZ
81.242%
13.662%
ratio=5.95
57.458%
34.436%
ratio=1.67
86.879%
9.777%
ratio=8.89
86.851%
8.723%
ratio=9.96
BL2-A
BL1-A

Figure S4

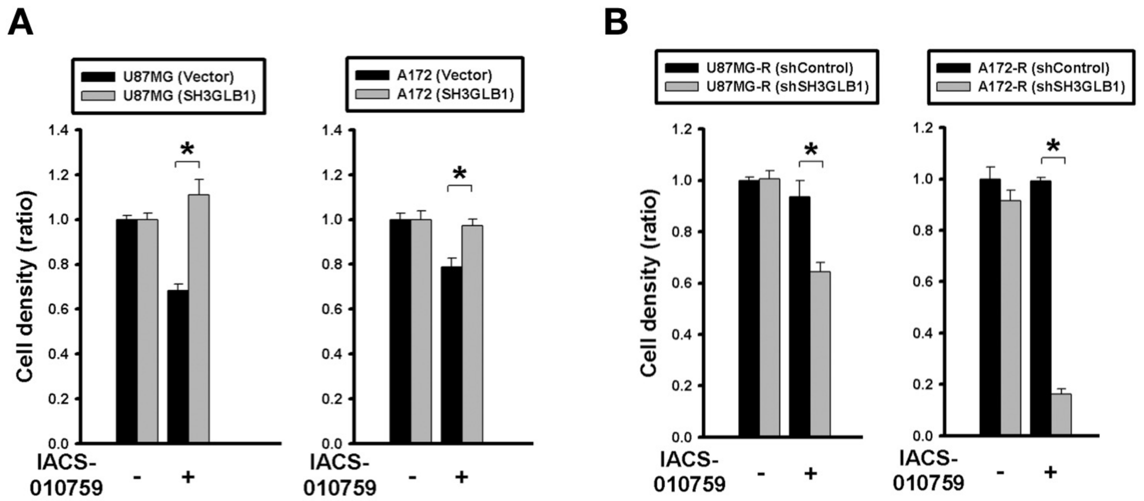
A
U87MG (Vector)
U87MG (SH3GLB1)
A172 (Vector)
A172 (SH3GLB1)
Cell density (ratio)
1.4
1.2
1.0
0.8
0.6
0.4
0.2
0.0
*
IACS-010759
-
+
B
U87MG-R (shControl)
U87MG-R (shSH3GLB1)
A172-R (shControl)
A172-R (shSH3GLB1)

Figure S5

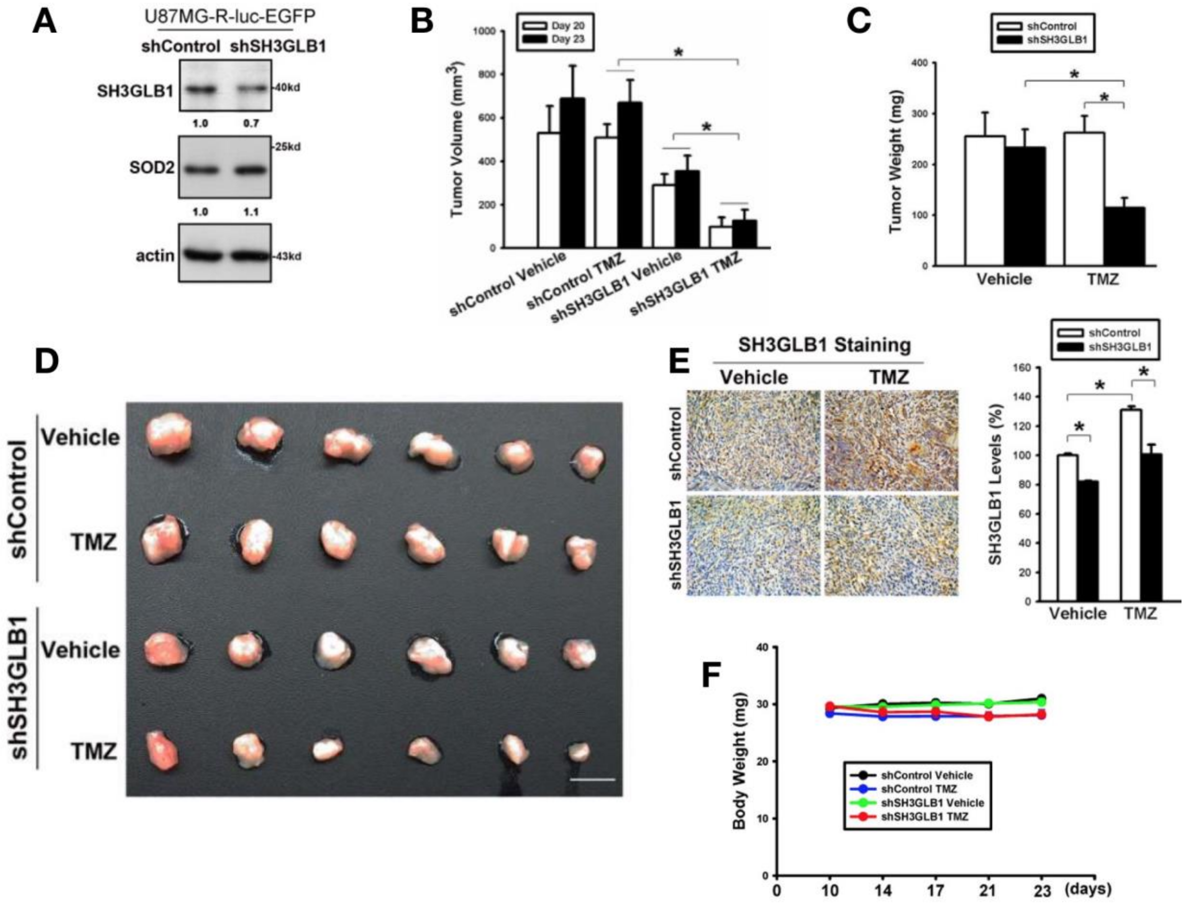
A
U87MG-R-luc-EGFP
shControl shSH3GLB1
SH3GLB1
40kd
1.0 0.7
SOD2
25kd
1.0 1.1
actin
43kd
B
Day 20
Day 23
Tumor Volume (mm3)
shControl Vehicle
shControl TMZ
shSH3GLB1 Vehicle
shSH3GLB1 TMZ
C
shControl
shSH3GLB1
Tumor Weight (mg)
Vehicle
TMZ
D
shControl
Vehicle
TMZ
shSH3GLB1
Vehicle
TMZ
E
SH3GLB1 Staining
Vehicle
TMZ
shControl
shSH3GLB1
SH3GLB1 Levels (%)
F
Body Weight (mg)
shControl Vehicle
shControl TMZ
shSH3GLB1 Vehicle
shSH3GLB1 TMZ
(days)

Figure S6.

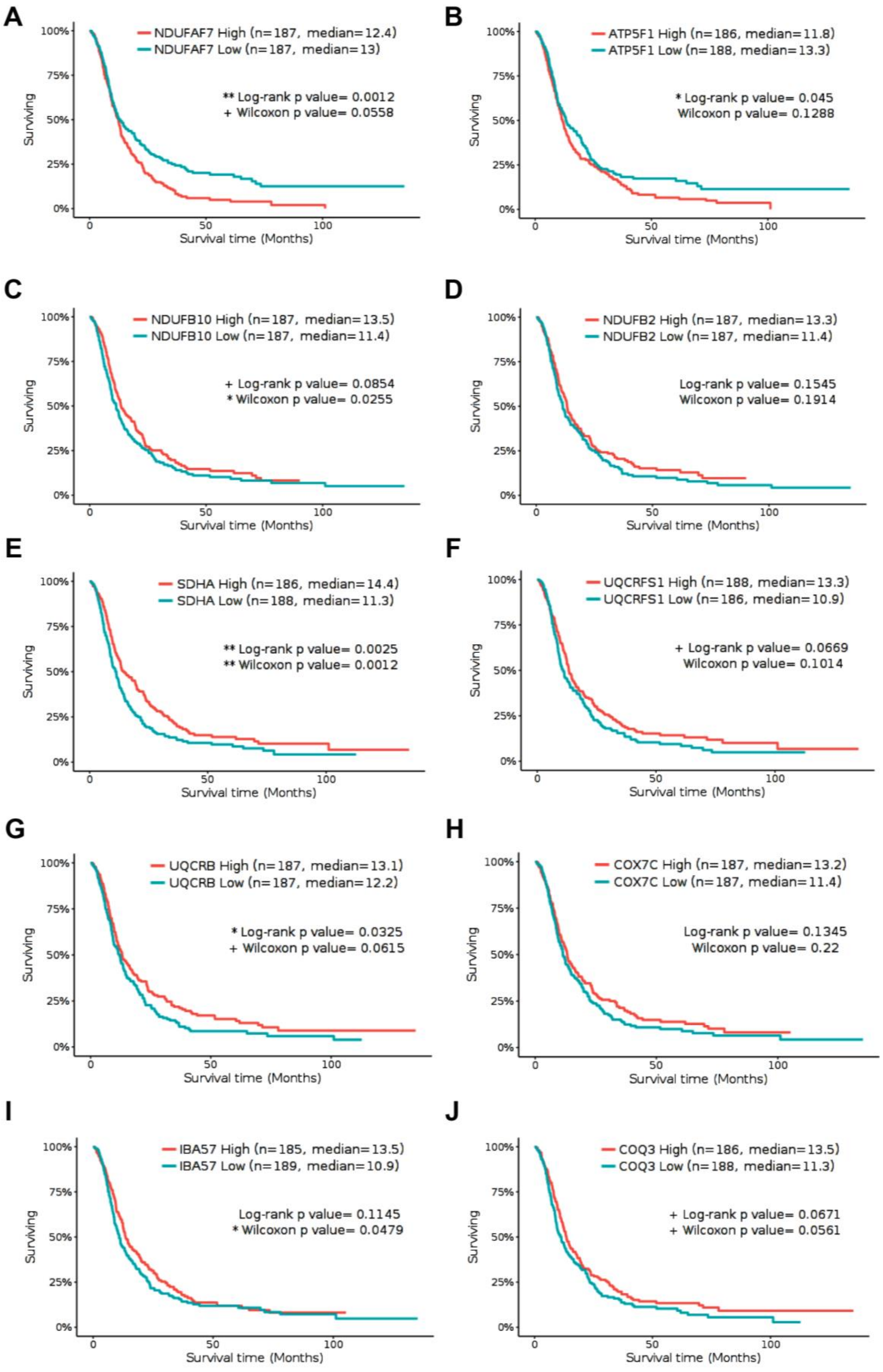
A
NDUFAF7 High (n=187, median=12.4)
NDUFAF7 Low (n=187, median=13)
** Log-rank p value= 0.0012
+ Wilcoxon p value= 0.0558
B
ATP5F1 High (n=186, median=11.8)
ATP5F1 Low (n=188, median=13.3)
* Log-rank p value= 0.045
Wilcoxon p value= 0.1288
C
NDUFB10 High (n=187, median=13.5)
NDUFB10 Low (n=187, median=11.4)
+ Log-rank p value= 0.0854
* Wilcoxon p value= 0.0255
D
NDUFB2 High (n=187, median=13.3)
NDUFB2 Low (n=187, median=11.4)
Log-rank p value= 0.1545
Wilcoxon p value= 0.1914
E
SDHA High (n=186, median=14.4)
SDHA Low (n=188, median=11.3)
** Log-rank p value= 0.0025
** Wilcoxon p value= 0.0012
F
UQCRFS1 High (n=188, median=13.3)
UQCRFS1 Low (n=186, median=10.9)
+ Log-rank p value= 0.0669
Wilcoxon p value= 0.1014
G
UQCRB High (n=187, median=13.1)
UQCRB Low (n=187, median=12.2)
* Log-rank p value= 0.0325
+ Wilcoxon p value= 0.0615
H
COX7C High (n=187, median=13.2)
COX7C Low (n=187, median=11.4)
Log-rank p value= 0.1345
Wilcoxon p value= 0.22
I
IBA57 High (n=185, median=13.5)
IBA57 Low (n=189, median=10.9)
Log-rank p value= 0.1145
* Wilcoxon p value= 0.0479
J
COQ3 High (n=186, median=13.5)
COQ3 Low (n=188, median=11.3)
+ Log-rank p value= 0.0671
+ Wilcoxon p value= 0.0561
Surviving
100%
75%
50%
25%
0%
Survival time (Months)
0
50
100

Figure S7

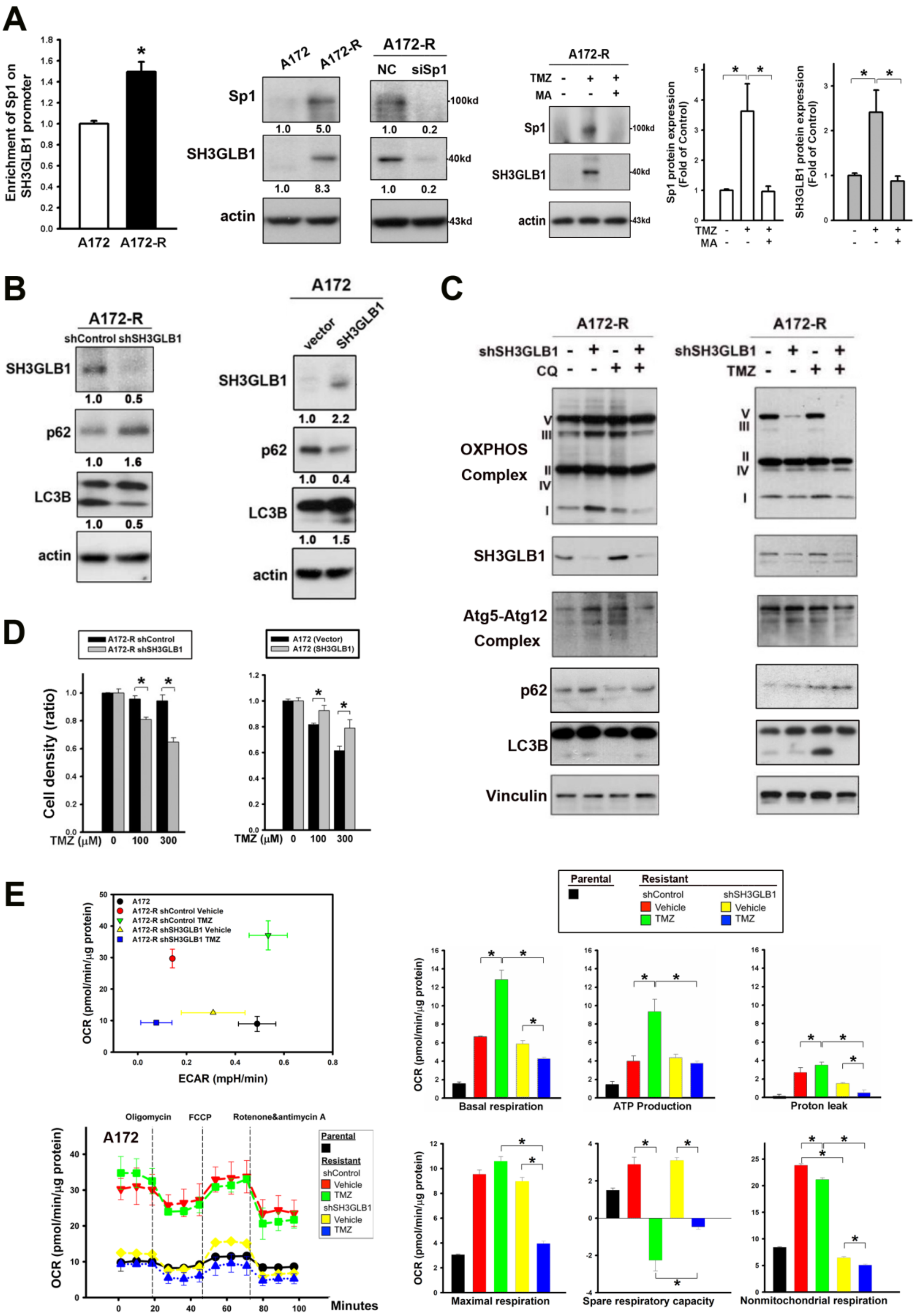
A
Enrichment of Sp1 on SH3GLB1 promoter
A172
A172-R
Sp1
SH3GLB1
actin
NC
siSp1
100kd
40kd
43kd
TMZ
MA
Sp1 protein expression (Fold of Control)
SH3GLB1 protein expression (Fold of Control)
B
shControl shSH3GLB1
p62
LC3B
vector
C
shSH3GLB1
CQ
OXPHOS Complex
Atg5-Atg12 Complex
Vinculin
D
A172-R shControl
A172-R shSH3GLB1
A172 (Vector)
A172 (SH3GLB1)
Cell density (ratio)
TMZ (μM)
E
A172-R shControl Vehicle
A172-R shControl TMZ
A172-R shSH3GLB1 Vehicle
A172-R shSH3GLB1 TMZ
OCR (pmol/min/μg protein)
ECAR (mpH/min)
Oligomycin
FCCP
Rotenone&antimycin A
Parental
Resistant
shControl
Vehicle
TMZ
shSH3GLB1
Minutes
Basal respiration
ATP Production
Proton leak
Maximal respiration
Spare respiratory capacity
Nonmitochondrial respiration

Figure S8

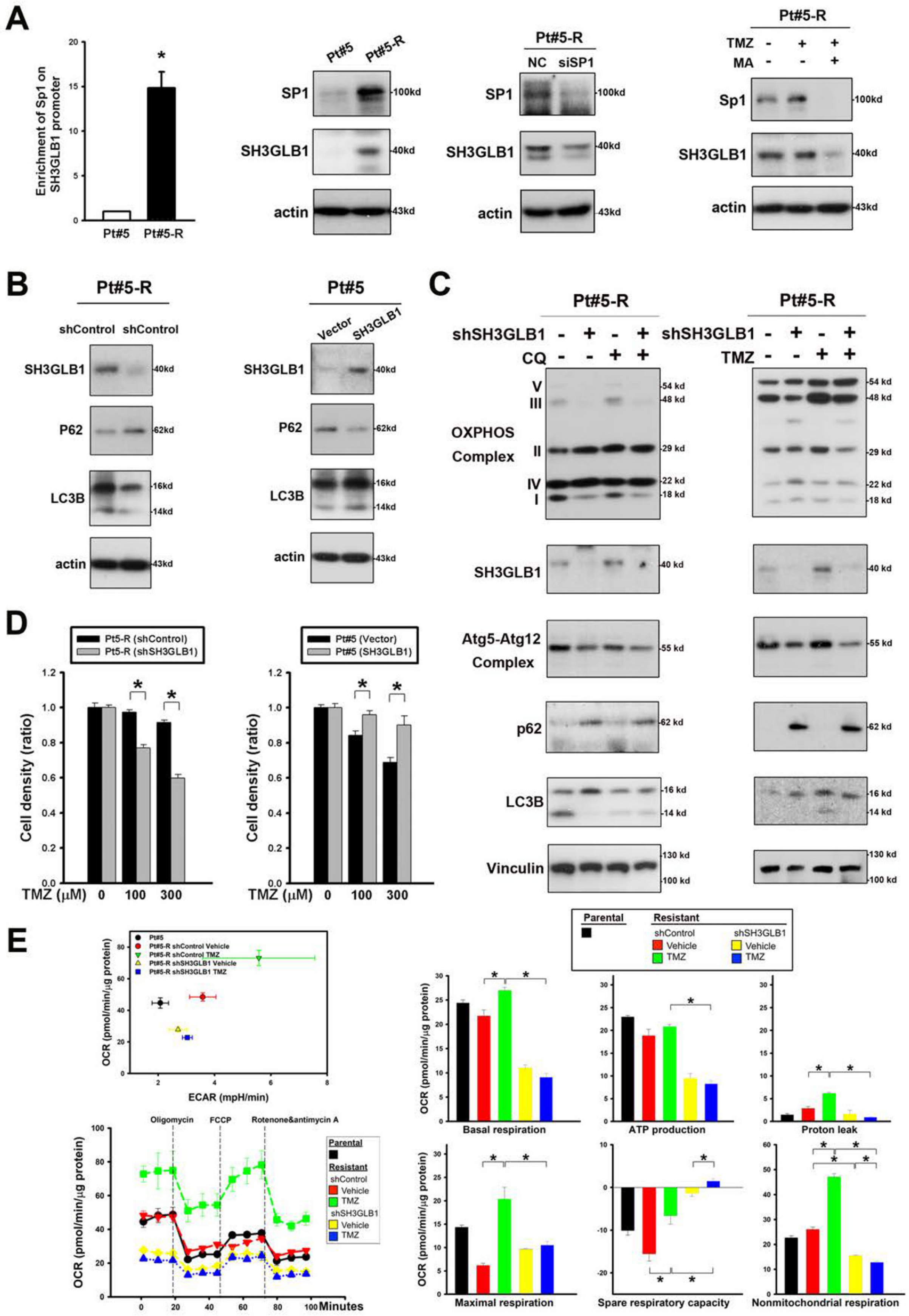

Figure S9
